# A benzopyran with antiarrhythmic activity is an inhibitor of Kir3.1-containing potassium channels

**DOI:** 10.1016/j.jbc.2021.100535

**Published:** 2021-03-11

**Authors:** Meng Cui, Yaser Alhamshari, Lucas Cantwell, Said EI-Haou, Giasemi C. Eptaminitaki, Mengmeng Chang, Obada Abou-Assali, Haozhou Tan, Keman Xu, Meghan Masotti, Leigh D. Plant, Ganesh A. Thakur, Sami F. Noujaim, James Milnes, Diomedes E. Logothetis

**Affiliations:** 1 Department of Pharmaceutical Sciences, School of Pharmacy, Bouvé College of Health Sciences, Northeastern University, Boston, Massachusetts, USA; 2 Department of Cardiac Biology, Xention Ltd, Cambridge, UK; 3 Department of Molecular Pharmacology & Physiology, Morsani College of Medicine, University of South Florida, Tampa, Florida, USA; 4 Center for Drug Discovery, Northeastern University, Boston, Massachusetts, USA; 5 Department of Chemistry and Chemical Biology, College of Science, Northeastern University, Boston, Massachusetts, USA

**Keywords:** ion channel, inhibition mechanism, homology modeling, molecular docking, molecular dynamics, AF, atrial fibrillation, benzopyran- (BP-) G1, (3R,4S)-7-(hydroxymethyl)-2,2,9-trimethyl-4-(2-phenylethylamino)-3,4-dihydropyrano[2,3-g]quinolin-3-ol, HBC, helix bundle crossing, HK, high potassium, I_KACh_, acetylcholine-activated inwardly rectifying K^+^ current, Kir3, G-protein-gated inwardly rectifying K^+^ channel subfamily 3, MD, molecular dynamics, TEVC, two electrode voltage-clamp

## Abstract

Atrial fibrillation (AF) is the most commonly diagnosed cardiac arrhythmia and is associated with increased morbidity and mortality. Currently approved AF antiarrhythmic drugs have limited efficacy and/or carry the risk of ventricular proarrhythmia. The cardiac acetylcholine activated inwardly rectifying K^+^ current (I_KACh_), composed of Kir3.1/Kir3.4 heterotetrameric and Kir3.4 homotetrameric channel subunits, is one of the best validated atrial-specific ion channels. Previous research pointed to a series of benzopyran derivatives with potential for treatment of arrhythmias, but their mechanism of action was not defined. Here, we characterize one of these compounds termed Benzopyran-G1 (BP-G1) and report that it selectively inhibits the Kir3.1 (GIRK1 or G1) subunit of the K_ACh_ channel. Homology modeling, molecular docking, and molecular dynamics simulations predicted that BP-G1 inhibits the I_KACh_ channel by blocking the central cavity pore. We identified the unique F137 residue of Kir3.1 as the critical determinant for the I_KACh_-selective response to BP-G1. The compound interacts with Kir3.1 residues E141 and D173 through hydrogen bonds that proved critical for its inhibitory activity. BP-G1 effectively blocked the I_KACh_ channel response to carbachol in an *in vivo* rodent model and displayed good selectivity and pharmacokinetic properties. Thus, BP-G1 is a potent and selective small-molecule inhibitor targeting Kir3.1-containing channels and is a useful tool for investigating the role of Kir3.1 heteromeric channels *in vivo*. The mechanism reported here could provide the molecular basis for future discovery of novel, selective I_KACh_ channel blockers to treat atrial fibrillation with minimal side effects.

Atrial fibrillation (AF), a common cardiac arrhythmia, affects over two million Americans and is associated with increased morbidity, mortality, and healthcare costs. Antiarrhythmic drugs (AADs) used to treat AF aim to either maintain the normal sinus rhythm (SR) or control ventricular rate (1). However, conventional drugs such as sodium channel blockers (class I antiarrhythmic drugs) and potassium channel blockers (class III antiarrhythmic drugs) have limited efficacy, especially in persistent AF patients. In addition, these conventional AADs carry a risk of ventricular proarrhythmia, such as torsade de pointes (TdP) through excessive delay of ventricular repolarization (2, 3). Thus, development of new antiarrhythmic agents with a more effective and safer profile is highly desirable (4).

The cardiac acetylcholine- (ACh-) activated inwardly rectifying K^+^ current (I_KACh_) consists of heterotetrameric ion channel subunits composed of Kir3.1 (GIRK1 or G1) and Kir3.4 (or GIRK4) subunits and is activated *via* the M2 muscarinic receptor to mediate vagal influences on heart rate and atrial repolarization. The Kir3.1/4 heterotetramers are the main contributors to the cardiac I_KACh_, while Kir3.4 homotetramers contribute considerably less activity. In contrast, Kir3.1 homotetramers are inactive and mostly trapped in the endoplasmic reticulum (ER) (5). Heteromerization with the Kir3.4 traffics the heterotetramer efficiently to the plasma membrane to contribute to I_KACh_. Mutation of Kir3.1(F137) to the corresponding Ser residue found in Kir3.4 produces functional Kir3.1(F137S) channels although their trapping in the ER continues (6, 7). This active Kir3.1 mutant has been an invaluable tool to assessing the role of Kir3.1 subunits in the regulation of heteromeric channel activity, but in addition, it has been found to be a critical determinant of activity in the action of urea-based channel activators (8, 9). Kir3 channel activity is strongly inwardly rectifying. Despite the relatively small outward conductance at physiological potentials, I_KACh_ is an important contributor to the late AP repolarization and plays a major role in stabilizing the resting membrane potential (10, 11). Activation of I_KACh_ has strong atrial AP-shortening and AF-promoting effects (12). Studies have shown that chronic AF alters I_KACh_ properties, by causing the channel to open even in the absence of ACh, resulting in APD shortening that promotes AF (13, 14, 15). In the heart, Kir3 channel subunits comprising I_KACh_ are prominently expressed in the sinus and atrioventricular nodes, as well as in the atrial myocardium but are largely absent in ventricles or contribute minimally to ventricular repolarization (16, 17). Therefore, targeting I_KACh_ channels is expected to selectively prolong the atrial effective refractory period (ERP) and therefore terminate AF without the risk of QT prolongation or TdP (1).

A number of I_KACh_ channel inhibitors have been developed in recent years. However, no selective I_KACh_ channel inhibitors have been approved for clinical use to date (11, 18). Tertiapin-Q is a nonoxidizable derivative of the naturally occurring peptide toxin tertiapin from the venom of the European honey bee (*Apis melifera*) that selectively inhibits Kir (Kir3 and Kir1 or ROMK) channels, as well as calcium-activated large-conductance potassium channels (BK) (19, 20). Tertiapin-Q selectively inhibits I_KACh_ channels in the cardiac tissue with high potency, without effect on Kir2.1 (IRK1) channels (21). Potential side effects of Tertiapin-Q could arise from inhibition of BK outward currents in the central nervous system (20), as well as immunogenicity and plasma protein binding in humans (22). NIP-142 was the first moderately selective I_KACh_ small-molecule blocker, which inhibited heterologously expressed Kir3.1/Kir3.4 and K_V_1.5 currents at low micromolar concentrations (23, 24). It terminates both AF in the canine vagal stimulation-induced AF model and atrial flutter in the canine Y-shaped incision-induced AF model (25). The follow-up compound NIP-151 has higher potency for I_KACh_ and selectivity over hERG channels, also effectively terminates AF in both vagal nerve stimulation-induced AF and aconitine-induced AF models. NIP-151 significantly prolonged the canine atrial ERP without significant effects on the ventricular ERP (26). NTC-801, a substituted benzopyran, was the first “selective” Kir3.1/Kir3.4 inhibitor investigated in clinical studies (27). It significantly prolonged the canine atrial ERP without affecting the ventricular ERP under vagal nerve stimulation. It effectively converted AF to normal sinus rhythm in both vagal nerve simulation-induced and the aconitine-induced AF model in dog and terminated and prevented the induction of AF in an atrial tachycardia-AF dog model of persistent AF (27, 28). However, a phase II study failed to meet the primary endpoint to reduce the AF burden in patients with paroxysmal AF. It is speculated that this was due to the relatively low dosing, which was necessary to avoid central nervous system side effects (1). In addition, I_KACh_ inhibitors, AZD2927 and A7071, did not affect atrial repolarization in atrial flutter patients (29). However, the lack of desirable effects of these compounds does not diminish the value of I_KACh_ channel inhibitors for persistent AF (1). Therefore, the need remains to develop new drug candidates selectively targeting I_KACh_ channels.

A group of benzopyran derivatives with triple rings was discovered having a prolongation effect on the atrial ERP, which can be used for treatment of arrhythmias. Conventional antiarrhythmic agents that use as their main mechanism of action prolongation of the refractory period (presumably due to prolongation of the action potential in ventricular muscle) present therapeutic problems; they induce highly dangerous arrhythmias leading to sudden death, such as torsades de pointes among others. In contrast, tricyclic benzopyran compounds selectively prolong the dog atrial ERP period without any influence on the ventricular ERP (30). However, the mechanism of their antiarrhythmic activity has been unclear. From this chemical series a selection patent (31) exemplifies a crystal form of a single 4-(aralkylamino)-2,2-dimethyl-3,4-dihydro-2H-benzopyran-3-ol compound. An undisclosed substitution of this compound is inferred to be the clinical candidate NTC-801 (27).

We synthesized the patented (31) tricyclic benzopyran compounds (enantiomers), (3R,4S)-7-(hydroxymethyl)-2,2,9-trimethyl-4-(2-phenylethylamino)-3,4-dihydropyrano[2,3-g]quinolin-3-ol and found one, BP-G1 (Fig. 1*A*) to selectively and potently inhibit heteromeric Kir3.1/4 or Kir3.1/2 channels (IC_50s_ ∼ 10–30 nM) over other cardiac channels, including homomeric Kir3 and Kir2 channels. The compound can be used as a useful pharmaceutical tool to explore the mechanism of action of tricyclic benzopyran compounds through their interactions with the I_KACh_/Kir3.x channels, as well as their selectivity over other ion channels. In this work, we characterized the molecular mechanism of BP-G1 action and its interaction with the Kir3.1/4 channel, using a combination of computational and experimental approaches.

**Figure 1:**
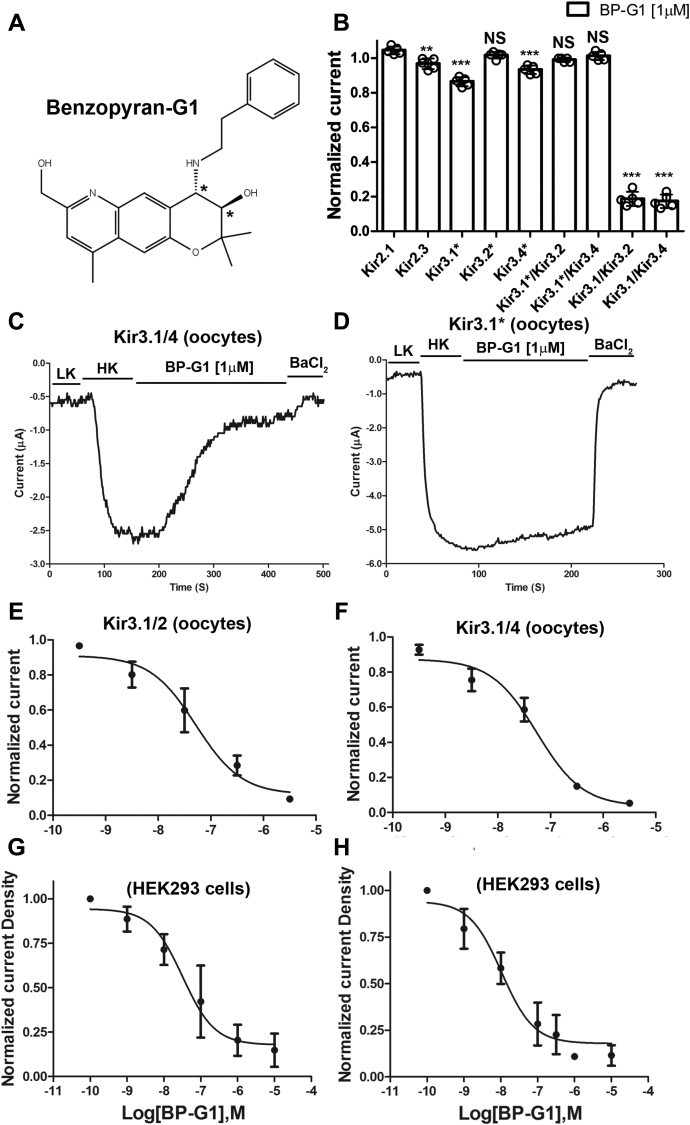
**Benzopyran-G1 is a potent inhibitor of GIRK1-containing heteromers.***A*, 2D structure of BP-G1. *Asterisks* mark chiral carbons. *B*, inhibitory activity of the BP-G1 compound [1 μM] on Kir channels. The basal current (normalized) is measured in a high potassium (HK) solution ND96K. *C*, representative traces of responses to the BP-G1 compound [1 μM] of Kir channels, Kir3.1/Kir3.4, and (*D*) Kir3.1∗. HK, high potassium solution ND96K; LK, low potassium solution ND96. *E*, concentration–response curves of the BP-G1 compound inhibition on the Kir3.1/2 (IC_50_ = 55.0 nM), and (*F*) Kir3.1/4 (IC_50_ = 55.9 nM) channels from TEVC. *G*, concentration–response curves of the BP-G1 compound inhibition on the Kir3.1/2 (IC_50_ = 32.2 nM), and (*F*). Kir3.1/4 (IC_50_ = 10.5 nM) channels from patch clamp. The *asterisks* indicate significant differences tested by one-way ANOVA Tukey’s test (∗∗∗*p* < 0.001,∗∗*p* < 0.01) compared with Kir2.1 (TEVC, data are mean ± SD, N = 5; Whole-cell patch clamp, N > 5).

## Results

### The BP-G1 compound selectively inhibits heteromeric Kir3 channels containing the Kir3.1 subunit

A group of benzopyran derivatives with triple rings was discovered to have a prolongation effect on the refractory period of atrial muscle without any influence on the refractory period and action potential of ventricular muscle (30). However, the mechanism of the antiarrhythmic activity of these drugs has been unclear. We tested whether benzopyran derivatives selectively inhibited Kir3.x channels that underlie cardiac I_KACh_ that is specifically found in the atrial myocardium. We tested the inhibitory activity of the benzopyran derivative BP-G1 (Fig. 1*A*) on several inwardly rectifier K^+^ channels, including Kir2.1, Kir2.3, Kir3.1∗, Kir3.2∗, Kir3.4∗, Kir3.1/2, and Kir3.1/4 channels by two electrode voltage-clamp (TEVC) (see Experimental procedures) experiments (where Kir3.1∗: Kir3.1(F137S); Kir3.2∗: Kir3.2(E152D); Kir3.4∗: Kir3.4(S143T)). The compound showed strong inhibitory selectivity for heteromeric Kir3 channels containing Kir3.1 (Kir3.1/2 and Kir3.1/4) over other Kir channels, including homomeric Kir3 channels (Kir3.1∗, Kir3.2∗, Kir3.4∗, Kir3.1∗/Kir3.2 and Kir3.1∗/Kir3.4) (Fig. 1*B*). Figure 1, *C* and *D* show representative experiments of responses of Kir3.1/4 and Kir3.1∗ channels to the compound. Upon application of the BP-G1 [1 μM], the Kir3.1/3.4 (or Kir3.1/4) channel current was significantly reduced over 100 s as compared with the Kir3.1∗ channel current. BP-G1 selectively inhibited heteromeric Kir3.1/2 and Kir3.1/4 whole-cell currents in a concentration-dependent manner and with comparable potency, IC_50_s of 55.9 nM and 55.0 nM in TEVC oocyte experiments (Fig. 1, *E* and *F*), and 32.2 nM and 10.5 nM in whole-cell patch-clamp HEK-293 cell experiments, respectively (Fig. 1, *G* and *H*).

### The BP-G1 compound inhibits the Kir3.4/4(S143F) channel mutant

**Figure 2:**
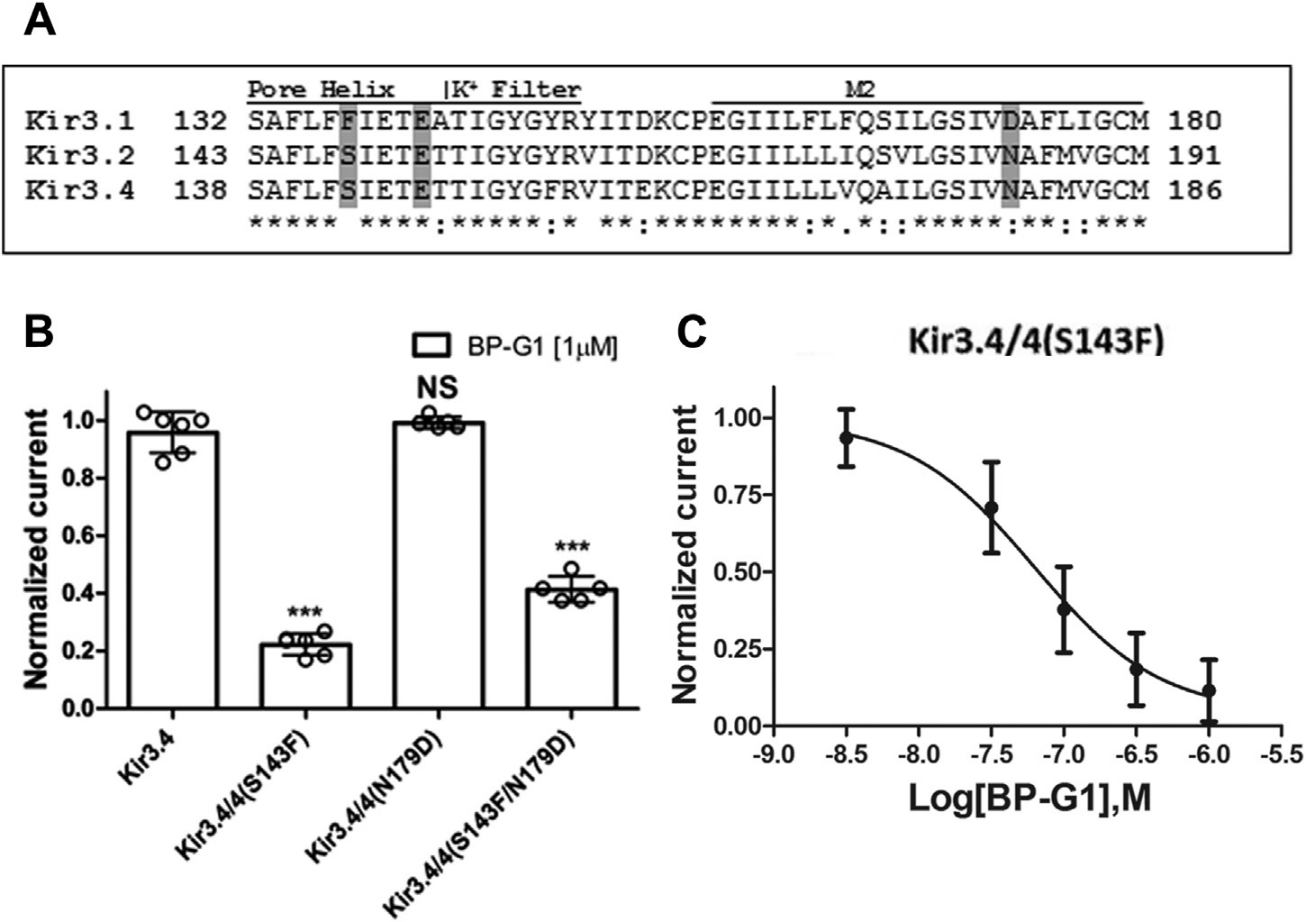
**F137 of Kir3.1 confers sensitivity to BP-G1 inhibition.***A*, sequence alignment among Kir3.1, Kir3.2, and Kir3.4 channels. Only the sequences of the Pore Helix, K^+^ Filter, and M2 helix regions are shown. “∗”: conserved, “:”: semiconserved, “.”: similar amino acids. The residues F137 and D173 in Kir3.1 and the corresponding residues in Kir3.2 and Kir3.4 are highlighted in *gray*. *B*, inhibitory activity of the BP-G1 compound [1 μM] to wild-type and mutated Kir3.4 channels to mimic Kir3.1 corresponding residues. The basal current (normalized) is measured in a high potassium (HK) solution ND96K. *C*, concentration–response curve of the BP-G1 compound inhibition on the Kir3.4/4(S143F) (IC_50_ = 62.1 nM). The *asterisks* indicate significant differences tested by one-way ANOVA Tukey’s test (∗∗∗*p* < 0.001) compared with Kir3.4 wild type (TEVC, data are mean ± SD, N ≥ 5).

The pore helix (F137) residue has been shown to be a critical Kir3.1 determinant for current potentiation of heteromeric Kir3 currents and also for ML297- and GAT1508-induced current potentiation (6, 7, 8, 9). In addition, the M2 helix residue D173 was also shown to be critical for ML297- and GAT1508-induced current stimulation (Fig. 2*A*). Thus, we proceeded to test whether Kir3.1(F137) and Kir3.1(D173) were also important for BP-G1 inhibition in the background of Kir3.4 channels. We chose to mutate the S143 and N179 corresponding residues of the Kir3.4 to the F137 and D173 of the Kir3.1 channel and coexpressed them with Kir3.4 wild-type subunits (6). Interestingly, while BP-G1 did not inhibit the wild-type Kir3.4 channel or the Kir3.4/4(N179D) channel mutant, it did inhibit the Kir3.4/4(S143F) channel mutant in a concentration-dependent manner (IC_50_ = 62.1 nM) (Fig. 2, *B* and *C*). In the background of the BP-G1 sensitive Kir3.4/4(S143F) channel, the double-mutant Kir3.4/4(S143F, N179D) showed a 20% less inhibition by BP-G1 (Fig. 2*B*). Because an Asp residue in position 179 in the context of the Kir3.4(S143F) did not contribute to the inhibition by BP-G1, the results of Figure 2 suggested that the pore helix Phe residue (S143F in Kir3.4 corresponding to F137 in Kir3.1 subunit) is a critical determinant for selective inhibition by BP-G1.

### Modeling the BP-G1 and Kir3.4 channel interactions and experimental validation

To better understand the molecular mechanism of the BP-G1 inhibition on Kir3 channels, we built homology models for the Kir3.4 and Kir3.1/4 channels based on a crystal structure of the Kir3.2 channel (PDBID: 3SYA). Sequence alignment among Kir3.1, Kir3.4, and Kir3.2 channels was generated by the ClustalW server (http://www.genome.jp/-tools/clustalw/). The sequence identities between Kir3.2 and Kir3.4 or Kir3.2 and Kir3.1 are 72% and 53%, respectively, which makes the Kir3.2 crystal structure an excellent structural template to generate accurate homology models for the Kir3.4, Kir3.1/2, and Kir3.1/4 channels (32). We used the MODELLER program (33) to generate ten initial homology models for Kir3.4 and Kir3.1/4, respectively, based on the Kir3.2 structural template and selected the one with the best internal DOPE (Discrete Optimized Protein Energy) score for modeling the compound and channel interactions.

**Figure 3:**
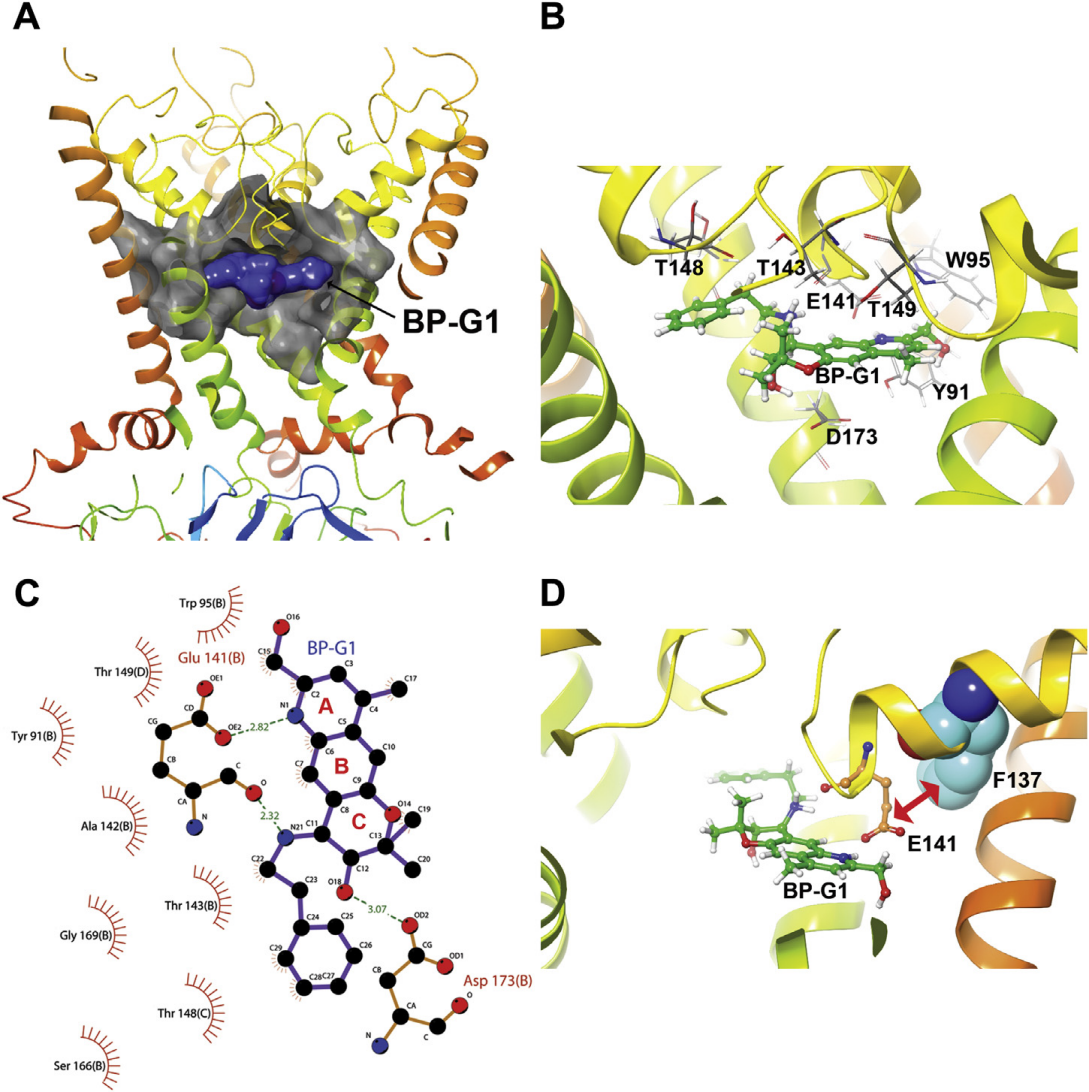
**Docking and predicted interactions of BP-G1 with a Kir3.1/4 model.***A*, predicted binding pocket in the Kir3.1/4 channel with docked BP-G1. *B*, BP-G1 interacts with the binding site residues of Kir3.1/4. *C*, the schematic depiction of the main interactions (in two dimensions) is shown for Kir3.1/4 with the BP-G1 compound (by LIGPLOT software). *D*, molecular model of Kir3.1/4 channel binding site with the docked BP-G1. The channel model is shown in *Ribbons* presentation. The BP-G1 and hydrogen-bonding interaction residue E141 (Kir3.1) are drawn in *ball* and *stick*, and residue F143 is shown in CPK representations. The *pink arrow* shows the residue E141 is repositioned by residue F137 in the Kir3.1 to interact with the BP-G1 compound through a salt bridge.

Based on the homology model of Kir3.1/4 channel, we performed molecular docking simulations to explore the potential binding site in the channel. Since our experimental results showed that the Kir3.1(F137) residue [corresponding to the Kir3.4(S143)] is critical for the inhibitory activity of BP-G1, we selected these residues together with the HBC residues F181(Kir3.1) and F187(Kir3.4) to define the grid box for docking simulations. The box covered the entire central cavity of the channel. We performed grid-based rigid protein and flexible docking using *Glide* and followed by Induced Fit Docking (*IFD*, Schrödinger, Inc), which takes into account the induced fit effects between the protein and ligand interactions. The pose with the lowest *XP* score from the *IFD* docking was selected as the predicted binding pose of the ligand (Fig. 3*A*). The detailed interactions between the BP-G1 and the channel were analyzed using the LIGPLOT program (34).

**Figure 4. Molecular determinants of BP-G1 inhibition:**
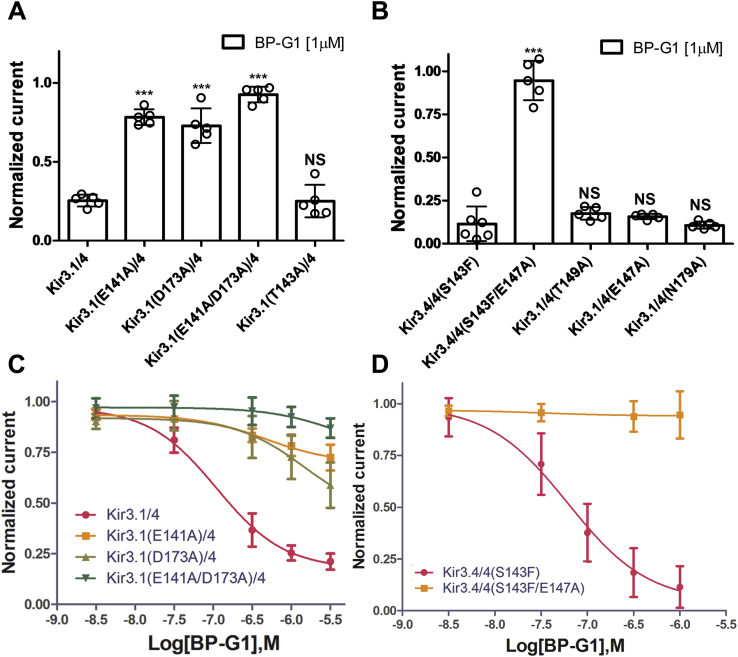
*A* and *B,* inhibitory activity of the BP-G1 compound [1 μM] on wild-type and mutated Kir3 channels. The basal current (normalized) is measured in a high potassium (HK) solution ND96K. *C* and *D*, concentration–response curve of the BP-G1 compound inhibition on the Kir3.1/4, Kir3.1 (E141A)/4, Kir3.1 (D173A)/4, and Kir3.1(E141A/D173A)/4 channels. The *asterisks* indicate significant differences tested by one-way ANOVA Tukey’s test (∗∗∗*p* < 0.001) compared with Kir3.1/4 or Kir3.4/4(S143F) (TEVC, data are mean ± SD, N ≥ 5).

Figure 3, *B* and *C* show predicted interactions between the BP-G1 and Kir3.1/4 channel. Three predicted interactions stood out: two between the nitrogen of ring A of the compound and a conserved residue in Kir3 channels (Kir3.1: E141 in the pore helix) and one between the hydroxyl of ring C of the compound and D173 that is unique to Kir3.1 compared with Asn residues found in the other Kir3 channels (32). Interestingly, the critical Kir3.1(F137) that is required for BP-G1 inhibition (Figs. 1*B* and 2, *B* and *C*) was not predicted by the model to interact directly with the compound. Instead, the model suggested that F137 influenced residue Kir3.1(E141), which directly interacted with the BP-G1 compound. Figure 3*D* shows the relationship of F137 to E141 relative to the compound. The bulky F137 side chain appears to enable sterically the salt bridge between E141 and the compound. To test the role of the Kir3.1(E141) residue in the BP-G1 inhibition, we mutated it to Ala. The Kir3.1(E141A)/4 showed only a 25% current block compared with the 75 to 80% block seen with the wild-type Kir3.1/4 (Figs. 1*B* and 4, *A* and *C*). In contrast, the neighboring conserved Kir3.1(T143A)/4 showed no significant reduction in BP-G1 block (Fig. 2*A*). We next probed the role of the Kir3.1 unique residue D173. The Kir3.1(D173A) mutant also showed a ∼25% current block by 1 μM BP-G1 (Fig. 4, *A* and *C*). Moreover, the double mutant Kir3.1(E141A/D173A) abolished any current block by BP-G1 (Fig. 4, *A* and *C*). As we saw in Figure 2, although BP-G1 showed no block of Kir3.4 currents, the single S143F mutant behaved as “Kir3.1-like” showing BP-G1 inhibition of the Kir3.4/4(S143F) heteromers. Based on the homology model of the Kir3.4 channel, we produced the Kir3.4/(S143F) heteromer model and performed molecular docking simulations to also explore the potential binding site of BP-G1 in this channel. As with the Kir3.1/4 model, the grid box included the S143F residue in the pore helix, the N179 residue in the TM2 together with the TM2 HBC residue F187 for docking simulations. The pose with the lowest *XP* score from the *IFD* docking was selected as the predicted binding pose of the ligand (Fig. S1*A*). The detailed interactions between the BP-G1 and the channel were analyzed using the LIGPLOT program (34).

Fig. S1, *B* and *C* shows predicted interactions between the BP-G1 and the Kir3.4/4(S143F) heteromer. Several similarities could be seen in comparing the Kir3.1/4 with the Kir3.4/4(S143F) model interactions with BP-G1. These included the E147 of the Kir3.4(S143F) subunit interaction with the nitrogen of ring A [similar to the corresponding Kir3.1(E141)] and the N179 interaction with the hydroxyl of ring C of the compound [similar to the corresponding Kir3.1(D173)]. Moreover, E141 of Kir3.1 or the corresponding E147 of the Kir3.4(S143F) subunit hydrogen bonded with the nitrogen of the phenylethylamino groupoff the dihydropyran ring C (Fig. S1*C*). We proceeded to test experimentally the predictions of the Kir3.4/4(S143F) with BP-G1. E147 was a dominant determinant for block by the compound, as the E147A mutant in the Kir3.4(S143F) subunit abolished block by the compound (Fig. 4, *B* and *D*). As we pointed out earlier, the N179D mutation in the Kir3.4(S143F) subunit did not result in greater inhibition, and if anything, it showed 20% less block than Kir3.4/4(S143F) (see Fig. 2*B*). These blocking effects of BP-G1 seemed to be exclusively taking place through the Kir3.1 [or Kir3.1-like, *i.e.*, Kir3.4(S143F)] subunit of the heteromer, as Kir3.4 mutants involving these residues, such as the E147A, N179A, or T149A, had no effect on the ability of BP-G1 to block heteromeric channel currents with Kir3.1 (Fig. 4*B*). Therefore, these experimental results validated the model predictions, indicating that the BP-G1 compound inhibits the Kir3.1/4 channel through the central pore cavity binding site formed predominantly by Kir3.1 residues E141 (through the F137 influence) and D173.

### Allosteric effects of BP-G1 on Kir3.1/4 channels

**Figure 5:**
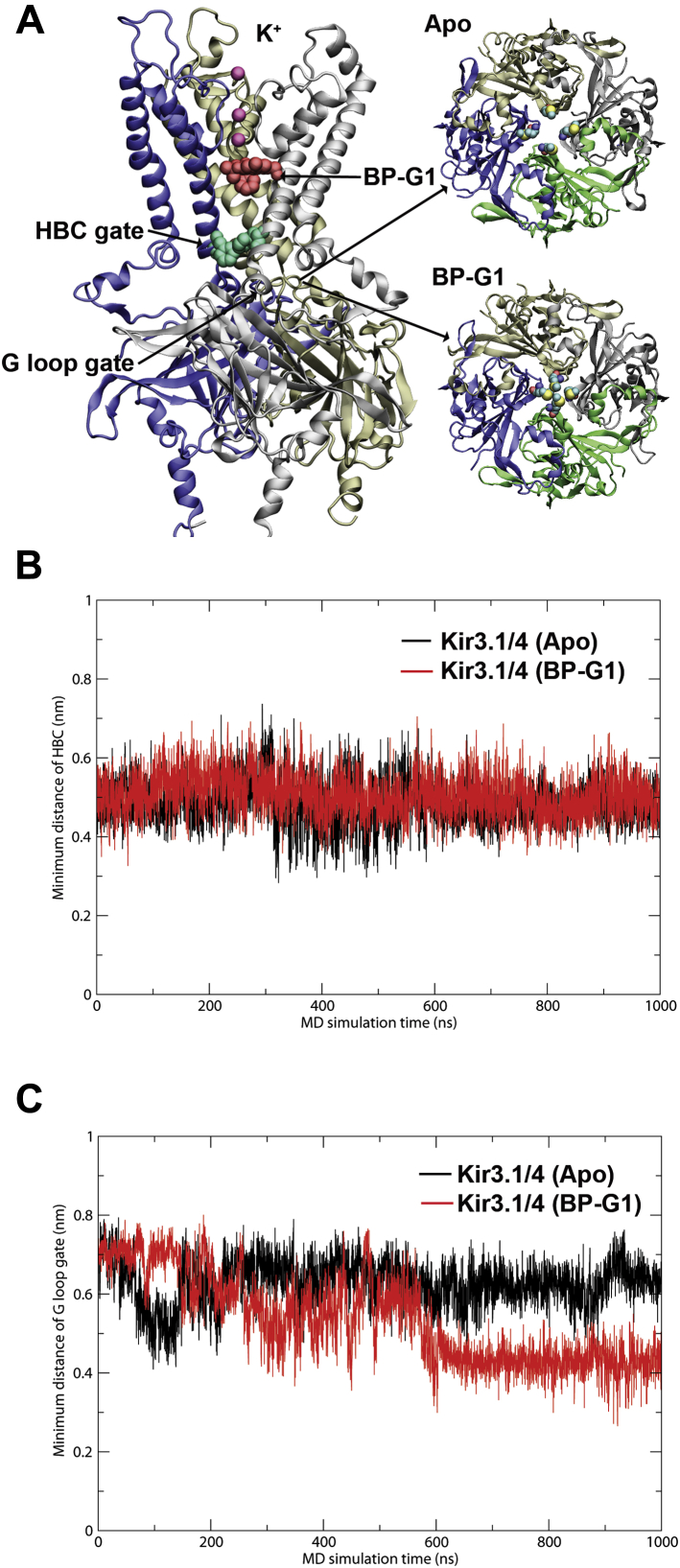
**MD simulation results on the Kir3.1/4-BP-G1 complex.***A*, *Left*, the snapshot of the complex after 1 μs simulations. BP-G1 (*red*), K^+^ ions, and HBC gate (*green*) are rendered in van der Waals spheres. The front subunit was removed for clarity. *Right*, *top view* of representative G loop gate from MD simulation trajectories, Apo (*upper*), BP-G1 bound (*lower*). Met residues are rendered in van der Waals spheres. *B*, minimum distance (averaged) between F181 (Kir3.1) and F187 (Kir3.4) as a function of the simulation time. *C*, minimum distance (averaged: T306, G307, M308, T309 (Kir3.1) and T312, G313, M314, T315 (Kir3.4)) of the G loop gate as a function of simulation time.

Even though our docking simulations and their experimental validations supported the model of BP-G1 acting as a pore blocker (Fig. 3*A* and Fig. S1*A*), we also performed molecular dynamics (MD) simulations asking whether additional allosteric effects were involved. We proceeded to perform 1μs MD simulations on Kir3.1/4 (Apo) and the predicted BP-G1-Kir3.1/4 complex in explicit lipid bilayer and water environments (Fig. S2*A*). The two systems reached equilibrium after about 100 ns of simulation (Fig. S2, *B* and *C*). Kir channels possess two gates, HBC (helix bundle crossing) and G loop, both of which need to be opened to allow potassium ions to pass through. Figure 5 shows the last snapshot from the MD simulations on the Kir3.1/4-BP-G1 system. BP-G1 occupied the central cavity of the channel and blocked ions from passing through. The minimum distances of the HBC gates in opposite subunits of Kir3.1/4/1/4 heterotetramers or Kir3.4 homotetramers (averaged distance measured between F181 (Kir3.1) or F187 (Kir3.4)) indicated they were closed (*i.e.*, 5.6 Å or less according to ref. (35)) for both Kir3.1/4 and Kir3.1/4-BP-G1 systems during the MD simulations (Fig. 5*B*). The G loop gate, after 500 ns, assumed a minimum distance in the Kir3.1/4-BP-G1 system that was shorter than that of Kir3.1/4 (Apo) (less than 5 Å), assuming an even tighter closed state (Fig. 5*C*). These studies suggest that BP-G1 besides occluding the permeation pathway, it may further stabilize allosterically the closed state of the G-loop gate of the Kir3.1/4 channel, thus further ensuring inhibition of channel activity.

### The BP-G1 enantiomer BP-G1(SR) does not inhibit heteromeric Kir3 channels

**Figure 6. Ring C modifications not tolerated for BP-G1 activity:**
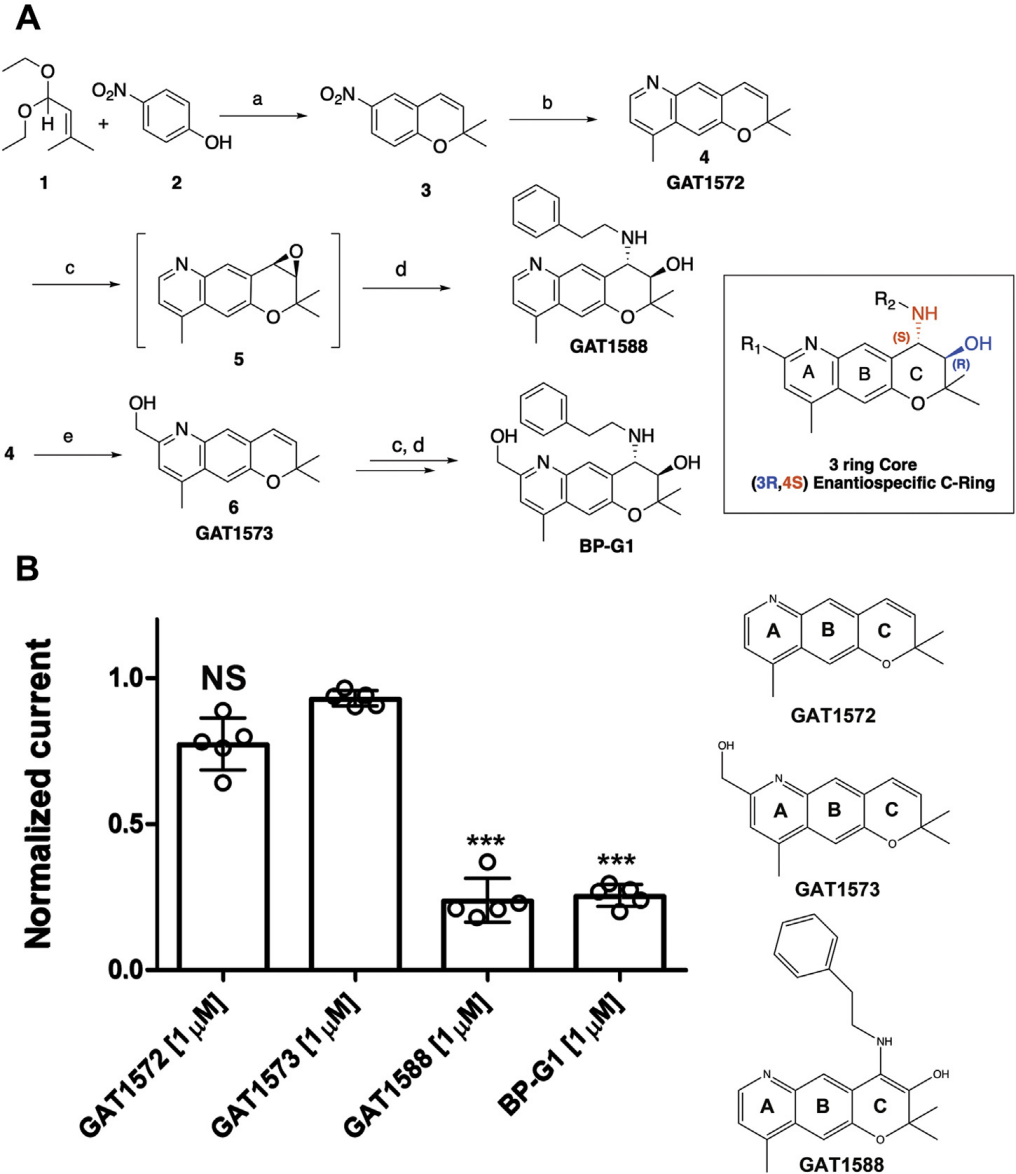
*A*, synthesis of the BP-G1 tricyclic core begins with the condensation of 11,diethoxy-3-methyl-2-butene (1) and 4-nitrophenol (2) to form the C-ring in compound 3. Subsequent nitro reduction followed by cyclization with methyl vinyl ketone results in formation of the A-ring shown in compound 4 (GAT1572). Chiral epoxidation of compound 4 followed with amination installs the enantiospecific centers on GAT1588. BP-G1 can be synthesized by starting with hydroxymethylation of compound 4. *B*, inhibitory activity of the GAT1572, GAT1573, GAT1588, and BP-G1 compounds [1 μM] to Kir3.1/4 channel. The ion channels were expressed in *Xenopus* oocytes and studied by TEVC. The basal current (normalized) is measured in a high potassium (HK) solution ND96K. The *asterisks* indicate significant differences tested by one-way ANOVA Tukey’s test (∗∗∗*p* < 0.001) compared with the GAT1573 (TEVC, data are mean ± SD, N = 5).

There are two chiral carbon atoms in the BP-G1 molecule (Fig. 1*A*, shown by asterisks). For comparison, we also docked the BP-G1 enantiomer (3S,4R)-7-(hydroxymethyl)-2,2,9-trimethyl-4-(2-phenylethylamino)-3,4-dihydropyrano[2,3-g]quinolin-3-ol [BP-G1] (BP-G1(SR)), which was unable to effectively inhibit the Kir3.1/2 or Kir3.1/4 channels (Fig. S3). BP-G1(SR) binds 180° flipped in the Kir3.1 binding pocket compared with its active (RS) enantiomer and could only form two hydrogen bonds [Kir3.1(E141) with the hydroxyl of ring C and Kir3.1(D173) with the hydroxyl of ring A] compared with BP-G1, which not only formed hydrogen bonds [Kir3.1(E141) with the hydroxyl rather than the nitrogen of ring A and Kir3.1(D173) with the hydroxyl of ring C] but also formed five hydrogen bonds with Kir3.1/4 channels (compare Fig. S3*B* and Fig. 3*C*). Moreover, the Kir3.1(D173) interaction with the hydroxyl of ring A is not functionally important, as its deletion (*i.e.*, in GAT1588) did not affect block of Kir3.1/4 currents (see Fig. 6*B*, next section). Thus, again the models proved consistent with the experimental results.

### Inhibitory activity of Kir3.1/4 currents by BP-G1 analogs

To gain insight as to the critical elements of the BP-G1 structure responsible for its inhibitory activity on Kir3.1/4 channel activity, three intermediate compounds of BP-G1 (GAT1572 (6: R^1^ = H, R^2^ = CH_3_), GAT1573 (6: R^1^ = CH_2_OH, R^2^ = CH_3_), and GAT1588 (1: R^1^ = H, R^2^ = CH_3_, R^3^ = (CH_2_)_2_-Ph)) were synthesized according to (Fig. 6*A*) and tested using TEVC (Fig. 6*B*). GAT1588 retained comparable inhibitory activity to BP-G1, suggesting that the hydroxyl of ring A was not a determinant of the effect but that changes to ring C were less tolerated making BP-G1 ineffective. These results were consistent with the modeling and mutagenesis results (Fig. 3 and Fig. S3), where no critical interactions were predicted with the hydroxyl of ring A but rather with the nitrogen of ring A and the hydroxyl of ring C as well as the nitrogen of the phenylethylamino group off the dihydropyran ring C, predictions that were supported by the mutagenesis results.

### BP-G1 is a highly specific inhibitor of Kir3.1-containing channels

**Figure 7:**
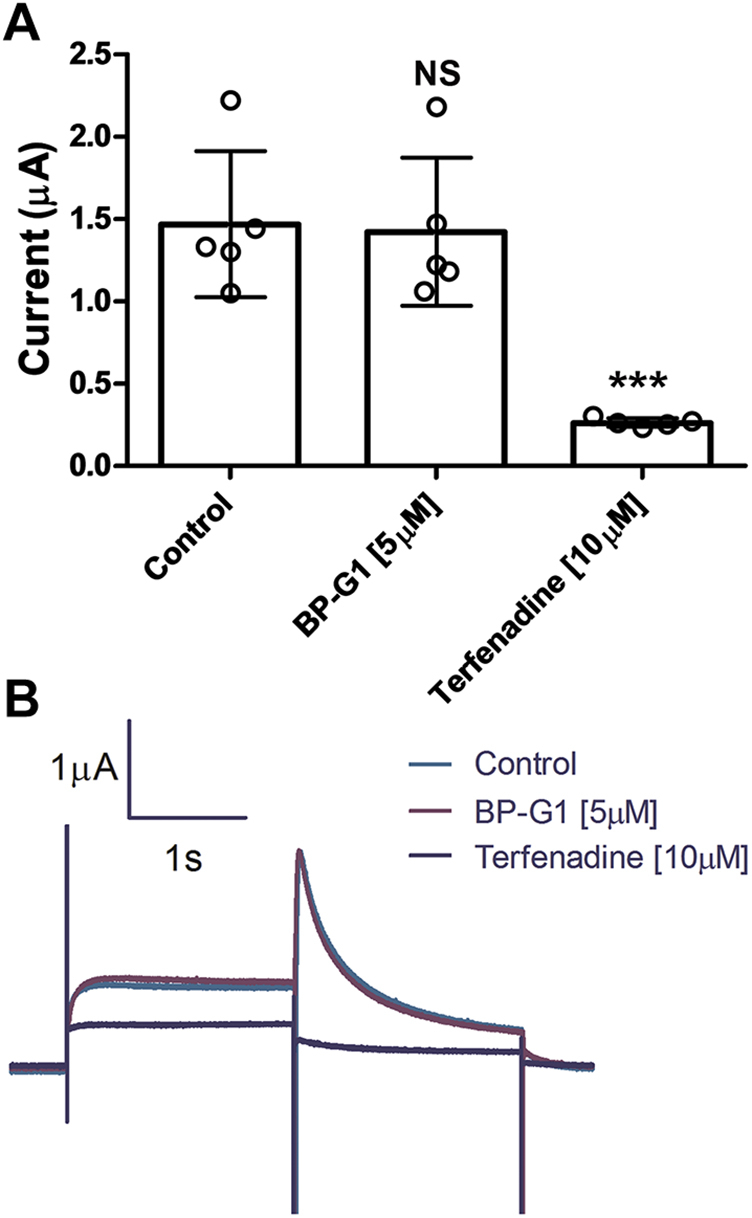
**hERG currents are not inhibited by BP-G1 [5 μM]**. hERG channels were expressed in *Xenopus* oocytes and studied by TEVC. hERG tail-current magnitude was assessed at −50 mV, following a 4 s test step to 20 mV from a holding voltage of −100 mV. The test pulse was repeated every 10-s. The residual current was inhibited by 10 μM terfenadine. *A*, bar graph of summary results. The *asterisks* indicate significant differences tested by one-way ANOVA Tukey’s test (∗∗∗*p* < 0.001) (data are mean ± SD N = 5). *B*, a representative trace of hERG channel currents (control, BP-G1, and Terfenadine).

BP-G1 effects were tested on hERG channel activity as required by FDA for any compound to remain on a drug candidate list. At the concentrations tested (up to 5 μM), BP-G1 did not show inhibitory activity on hERG current that was sensitive to block by terfenadine in heterologous expression recordings in *Xenopus oocytes* (Fig. 7). The inhibitory activity of BP-G1 was compared with published results for NTC-801 on several cardiac ion channels and are listed on Table S1. In addition, the inhibitory activity of BP-G1 [10 μM] and BP-G1(SR) [10 μM] was also tested on 80 protein targets using a radioligand binding assay (Tables S2 and S3, and Fig. S4). Three protein targets, the rat brain Na_V_ (65.1%), the dopamine transporter (91.2%), and the α1 adrenergic receptor (55.6%), exhibited BP-G1 binding. IC_50_s values of BP-G1 for the α1 adrenergic receptor and the dopamine transporter were 13 μM and 1 μM, respectively (Table S4). The rat brain Na_V_ channels did not distinguish between BP-G1 and BP-G1(SR) binding. Nevertheless, we tested in *Xenopus* oocytes one of the voltage-gated sodium channels most abundantly expressed in brain, Na_V_1.2, and found that 10 μM BP-G1 did not block these TTX-sensitive currents (Fig. S5).

### *In vivo* effects of *BP-G1*

**Figure 8:**
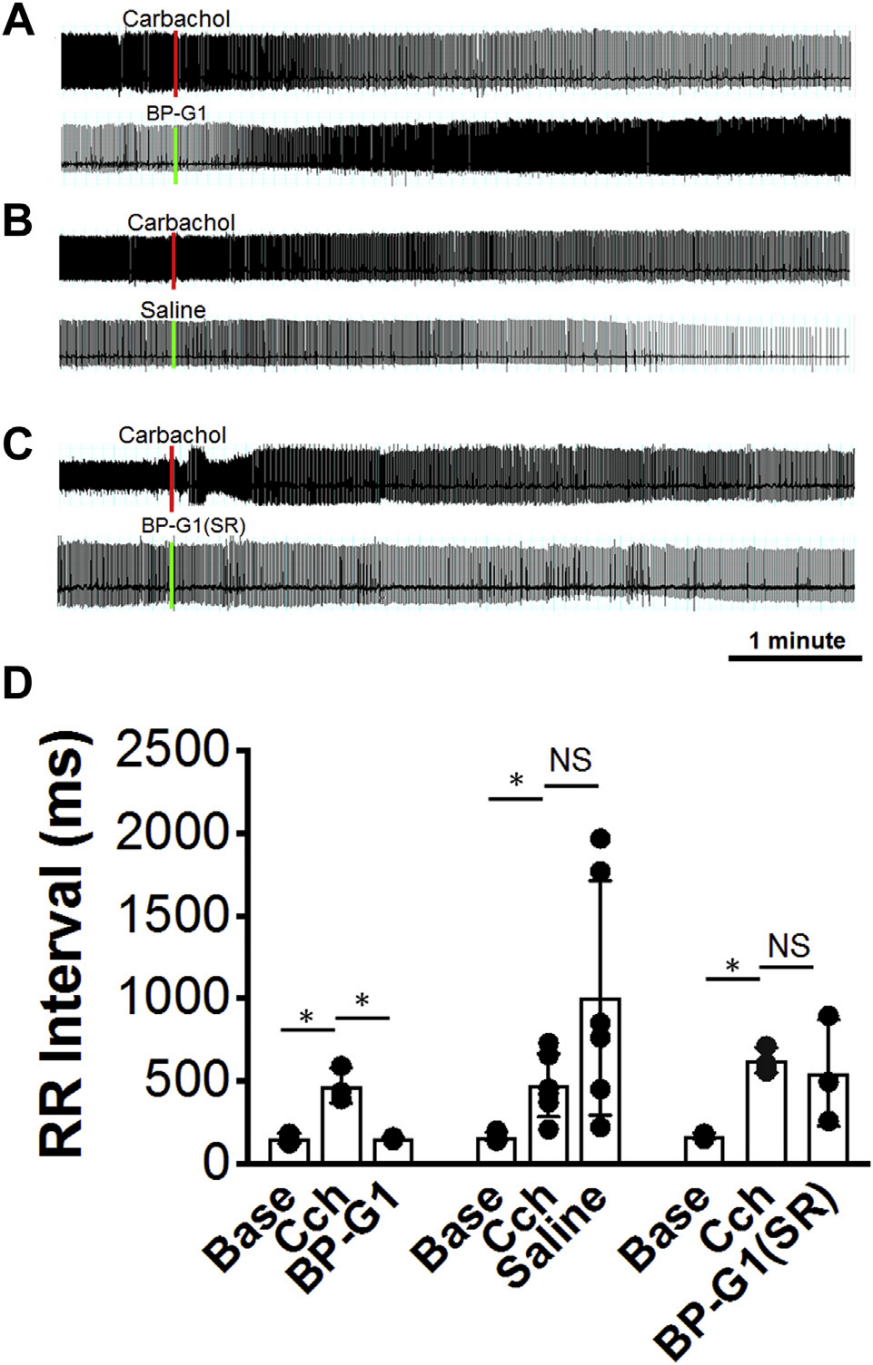
***In vivo* electrophysiological testing of BP-G1 effects on heart rate.** ECGs recorded from anesthetized mice and agents applied *via* injection through the jugular vein. *A*, carbachol injected first (*red line*) followed ∼10 min later by BP-G1 (*green line*) at concentrations given in the text. *B*, negative control experiment for (*A*), where Carbachol was again injected first (*red line*) followed ∼10 min later by Saline (*green line*). *C*, carbachol injected first (*red line*) followed ∼10 min later by BP-G1 (SR) (*green line*) at concentrations given in the text. *D*, *Left bars*, the baseline RR interval was 155.2 ± 16.3 ms. Carbachol caused a significant prolongation of the RR interval (474.2 ± 60.7 ms). Within 5 min following BP-G1, the RR interval shortened to baseline value (155.4 ± 5.5 ms) (∗*p* < 0.05, carbachol *versus* baseline and BP-G1). Repeated measures one-way ANOVA with Dunnett correction. *Middle bars*, the baseline RR interval was 168.6 ± 10.7 ms. Carbachol caused a significant prolongation of the RR interval (476.7 ± 77.8 ms). Five minutes after saline injection, the RR continued to prolong to 1005 ± 289 ms (∗*p* < 0.05, carbachol *versus* baseline and saline. Repeated measures one-way ANOVA with Dunnett correction). *Right bars*, the baseline RR interval was 146.1 ± 2.9 ms. Carbachol caused a significant prolongation of the RR interval (170.6 ± 10.1 ms). Five minutes after BP-G1 (SR) injection, the RR continued to prolong to 551.6 ± 185 ms (∗*p* < 0.05, carbachol *versus* baseline and saline. Repeated measures one-way ANOVA with Dunnett correction) (Bar graphs represent as mean ± SD. N = 3 animals for the BP-G1, N = 6 animals for the saline, and N = 3 animals for the BP-G1(SR) experiments).

We tested the *in vivo* effects of BP-G1 *versus* those of BP-G1(SR) on heart rate in anesthetized adult mice (Fig. 8). ECG was continuously recorded. Panel A is the ECG showing that after carbachol (300 μl of 200 μM) was administered, i.p. (red mark), the heart decelerated, with a visible RR prolongation. About 10 min after carbachol injection, BP-G1 was delivered *via* the jugular vein (40 μl of 240 μM) (green mark). Within 5 min, there was a reversal of carbachol’s effects on the heart rate. Panel B is a control mouse showing heart rate slowing after carbachol administration (red mark). But when 40 μl saline control was delivered through the jugular vein (green mark), the heart rate continued to slow down and did not recover. In Panel C we tested the inactive enantiomer BP-G1(SR). The ECG showed prolonged RR interval rate after carbachol administration (red mark). However, when 40 μl of the inactive form of BP-G1(SR) was injected through the jugular vein (green mark), the heart rate did not recover to baseline as was the case for BP-G1 shown in panel A.

Figure 8*D* is a quantification of RR interval in three mice treated with BP-G1 (*left bars*), six mice treated with saline control (*middle bars*), and three mice treated with BP-G1(SR) (*right bars*). In BP-G1, the baseline RR interval was 155.2 ± 16.3 ms. Carbachol caused a significant prolongation of the RR interval (474.2 ± 60.7 ms) because of muscarinic-mediated I_KACh_ activation by carbachol. Within 5 min after BP-G1, the RR interval shortened to the baseline value (155.4 ± 5.5 ms), due to block of I_KACh_ by BP-G1, thus abrogating the effects of carbachol on the RR interval (∗*p* < 0.05, carbachol *versus* baseline and BP-G1. Repeated measures one-way ANOVA with Dunnett correction). In saline control, the baseline RR interval was 168.6 ± 10.7 ms. Carbachol caused a significant prolongation of the RR interval (476.7 ± 77.8 ms). Five minutes after saline injection, the RR continued to prolong to 1005 ± 289 ms (∗*p* < 0.05, carbachol *versus* baseline and saline. Repeated measures one-way ANOVA with Dunnett correction). In BP-G1(SR), the baseline RR interval was 170.6 ± 10.1 ms. Carbachol caused a significant prolongation of the RR interval (627.6 ± 44.6 ms). Five minutes after BP-G1(SR), the RR did not shorten back to baseline as with BP-G1 and remained prolonged at 551.6 ± 185 ms (∗*p* < 0.05, carbachol *versus* baseline and saline. Repeated measures one-way ANOVA with Dunnett correction). This experiment clearly shows that BP-G1 is an active *in vivo* blocker of I_KACh._

## Discussion

Benzopyran derivatives with triple rings, such as BP-G1, have been shown to exert prolongation of the action potential refractory period selectively in atrial muscle. No influence of these derivatives was seen on the refractory period and action potential in ventricular muscle, making them potential antiarrhythmic drug candidates to treat AF with no ventricular side effects (30). However, the molecular mechanism of the drug’s action has not been clear. We tested BP-G1, a prototypic benzopyran derivative on various ion channels expressed in *Xenopus laevis* oocytes and mammalian HEK293 cells, using electrophysiology. We found that BP-G1 (Fig. 1 and Table S1) selectively inhibited Kir3.1 heteromeric currents (Kir3.1/4: IC_50_ = 10.5 nM, Kir3.1/2: IC_50_ = 32.2 nM) over other inwardly rectifying K^+^ channels and cardiac channels at large, such as homomeric Kir3.1∗, Kir3.4, Kir2.1, Kir2.3, hERG, Na_V_1.5, Kir6.2, Kir6.1, K_V_1.5, K_V_4.3, and K_V_1.7 channels. Interestingly, BP-G1 could only inhibit Kir3 channel heteromers containing the Kir3.1 subunit, namely Kir3.1/2 and Kir3.1/4, but not Kir3 channel homomers. Similar to ML297 (or GAT1508 for Kir3.1/2), which activates only Kir3.1-containing channels, BP-G1 is a unique inhibitor only of Kir3.1-containing channels. Previous studies showed that residues F137 and D179 in the Kir3.1 channel were critical for activation by ML297 and GAT1508. The double mutant Kir3.2:S148F/N184D + Kir3.2 WT could be activated by ML297 and GAT1508 (8, 9), demonstrating that these two residues of Kir3.1 are necessary and sufficient for activation by these drugs. We tested the single Kir3.4:S143F, N179D and double Kir3.4:S143F/N179D mutants coexpressed with Kir3.4 and found that the Kir3.1 (F137) was necessary and sufficient for the selective inhibition of heteromeric GIRK channels by the BP-G1 compound (Fig. 2).

To understand the molecular interactions between BP-G1 and Kir3.1/4, we built a homology model of Kir3.1/4 and docked BP-G1 into the central cavity of the channel binding site, which was identified by the CASTp program server (http://sts.bioe.uic.edu/castp). Although BP-G1 itself does not interact with F137 directly, F137 repositions the side chain of residue E141 to form two hydrogen bonds with the compound. To test the model prediction, we mutated the predicted interacting Kir3.1 residues E141 and D173 to Ala. Each of the single mutants showed a ∼25% block by BP-G1, while the double mutant abolished the block (Fig. 4). Thus, like ML297, the Kir3.1 residues F137 and D173 contribute critically to the BP-G1 effect on Kir3.1-containing heteromers. For BP-G1 F137 works indirectly through E141.

To explore the minimal structure fragment of BP-G1 required for activity, we synthesized three variants modifying mainly ring A (GAT1588) or C (GAT1572, GAT1573) (Fig. 6*A*). The compounds GAT1572 and GAT1573 were not active in inhibiting Kir3.1/4 channel activity. In contrast, GAT1588 inhibited the channel with comparable activity to BP-G1 (Fig. 6). Therefore, the R_2_ group or ring C is critical for the inhibitory activity of the compounds. The R_1_ group could be used to diversify the compound in further efforts to improve potency and selectivity. The medicinal chemistry results are in agreement with the modeling and mutagenesis results, which show no residue interactions with the hydroxyl of ring A, but residue interactions with the hydroxyl and nitrogen associated with ring C.

**Figure 9:**
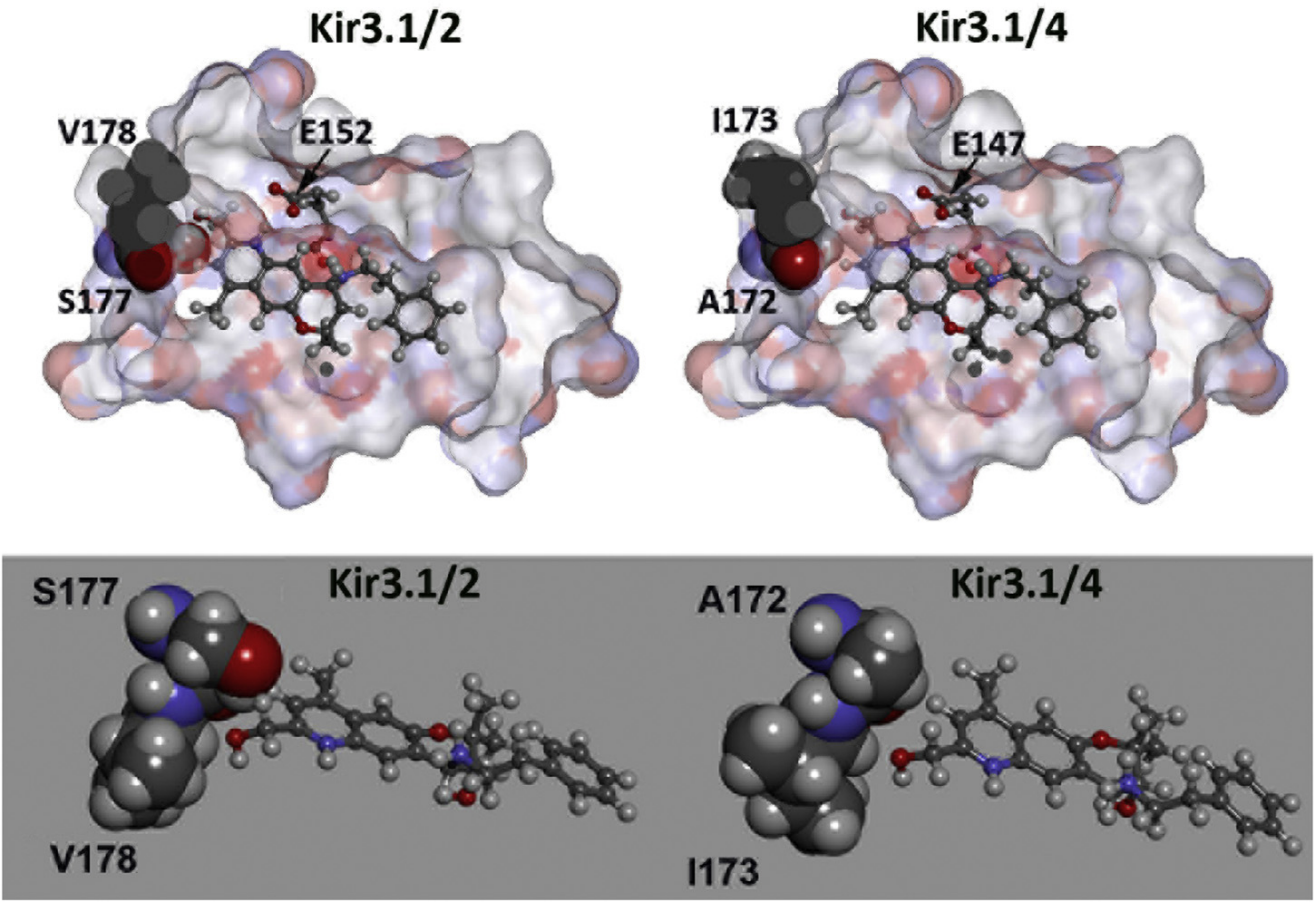
**Docking of BP-G1 reveals key amino acid (Kir3.2: S177, V178 and Kir3.4: A172, I173) differences between Kir3.2 and Kir3.4 that allows for structural variations proposed to be used to probe subtype selectivity of future designed compounds**.

Although BP-G1 exhibits very good inhibitory activity for I_KACh_, it also blocks Kir3.1/2 channels with high potency. Kir3.1/2 channels are highly expressed in neurons (36), therefore, blocking these channels could cause side effects. Improvement in the selectivity of BP-G1 for Kir3.1/4 channels over Kir3.1/2 and other off-targets would thus be highly desirable. By comparing the binding sites of Kir3.1/4 and Kir3.1/2 channel for BP-G1, only two residues are not conserved. A172 and I173 in Kir3.4 correspond to residues S177 and V178 in Kir3.2, respectively. Figure 9 shows the docked modes for the Kir3.1/2-BP-G1 and Kir3.1/4-BP-G1 complexes. The two-residue difference between Kir3.2 and Kir3.4 could be exploited to design BP-G1 analogs to improve the selectivity of inhibition for Kir3.1/4 over Kir3.1/2 channels. For example, possibly by optimizing the R_1_ group (Fig. 6*A*), Kir3.1/4 selective BP-G1 analogs could be obtained.

The compound BP-G1 has favorable properties as a drug. Predicted “Human Oral Absorption” was 81 (where >80% is considered very good). The predicted apparent MDCK cell permeability (QikProp, Schrödinger) was 36.4 nm/s. MDCK cells are a good model for blood–brain barrier permeability suggesting that BP-G1 will have moderate-to-low brain permeability. The BP-G1 also showed no inhibitory effect on hERG channels, as well as other cardiac ion channels such as Na_V_1.2, Na_V_1.5, Kir6.2, Kir6.1, K_V_1.5, K_V_4.3, and K_V_1.7 channels (Fig. 7, Fig. S5 and Table S1). Our rodent animal model suggested that BP-G1 is an active *in vivo* blocker of I_KACh_ (Fig. 8). In addition, *in vivo* ADME and pharmacokinetics were conducted (Table S5) substantiating the favorable predictions. Taken together, the BP-G1 could be a potential drug candidate for AF treatment; however, the selectivity of the compound would need to be further improved.

## Experimental procedures

Animal experiments were approved by the Institutional Animal Care and Use Committee at the University of South Florida, Tampa.

### Electrophysiology experiments

#### Two-electrode voltage clamp

*X. laevis* oocytes were isolated and microinjected as previously described (6). Human Kir3.1, mouse Kir3.2, and human Kir3.4 DNA constructs were used for electrophysiology experiments. All cRNA constructs were injected in the amount of 2 ng per oocyte. Injected oocytes were incubated for 2 to 3 days at 18 °C to allow for expression. Whole-cell oocyte currents were then measured using a GeneClamp 500 amplifier (Axon Instruments). Microelectrodes had resistances of 0.5 to 1 megohm using a 3M KCl solution in 1.4% agarose. Oocytes were perfused with a low K^+^ (LK) solution ND96 (2 mM KCl, 96 mM NaCl, 1 mM MgCl_2_, 1.8 mM CaCl_2_, 5 mM HEPES-Na) to establish a baseline for the recordings. Basal current was measured in a high potassium (HK) solution ND96K (96 mM KCl, 10 mM HEPES-K, 1 mM MgCl_2_, 1.8 mM CaCl_2_). To block the current, the oocyte chamber was perfused with 4 mM BaCl_2_ in ND96K. Only the Barium-sensitive current was used for statistical analysis. To measure the inhibitory activity of the channels, 1 μM BP-G1 in ND96K (HK) was used. Typically, oocytes were held at 0 mV (E_K_) and the current was monitored constantly using a ramp protocol with a command potential from −80 to +80 mV. Current amplitude was measured at the end of a 1 s sweep. All currents were analyzed when they reached steady state. Error bars in the figures represent SD (N > 4).

#### Maintenance of ion channel expressing cell lines

For the experiments shown in Figure 1, *G* and *H*, whole-cell currents were recorded with an Axopatch 200B amplifier and pCLAMP software (Molecular Devices). HEK293T cells were transiently transfected with 1 μg Kir3.1 together with 1 μg Kir3.2 or 1 μg Kir3.4 DNA, using polyethylenimine (PEI). The cells were maintained in DMEM supplemented with 10% Fetal Bovine Serum and 1% Penicillin/Streptomycin at 37 °C in a 5% CO_2_ humidified atmosphere and were studied 24 to 48 h after transfection. HEK293T cells were bathed in Low K^+^ (LK) solution (135 mM NaCl, 5 mM KCl, 1.2 mM MgCl_2_, 1.5 mM CaCl_2_, 8 mM Glucose, 10 mM HEPES with pH 7.4). High K^+^ (HK) solution (5 mM NaCl, 135 mM KCl, 1.2 mM MgCl_2_, 1.5 mM CaCl_2_, 8 mM Glucose, 10 mM HEPES with pH 7.4) was perfused to establish basal currents. BP-G1 in HK solution was perfused sequentially in a concentration response manner prior to applying 5 mM BaCl_2_ in HK to identify remaining barium-insensitive leak currents and subtract them from the current records. The experiments were performed at room temperature and the recording electrodes had resistances ranging from 2 to 4 megohms, when filled with internal solution (140 mM KCl, 2 mM MgCl_2_, 1 mM EGTA, 5 mM Na_2_ATP, 0.1 mM Na-GTP, 5 mM HEPES with pH 7.4). The whole-cell capacitance of HEK293T cells was 7 to 12 pF and the series resistance was typically <5 megohms. Cells used in the experiments were selected based on eGFP fluorescence. A ramp protocol from −80 mV to +80 mV was used and currents were recorded at −80 mV in the whole-cell mode (using the Strathclyde Electrophysiology Software from the University of Strathclyde, Glasgow). The patch-clamp data were analyzed offline in pClamp (Molecular Devices) and OriginLab software. The channel blocking, after application of BP-G1, was normalized based on the HK current, with the HK current representing 100% activity of the channel. At least five recordings were obtained from HEK293T cells at different passages, in each group. Error bars represent S.E.M.

For the data shown in Table S6, HEK293 cells expressing Kir3.1/4, Kir6.2/SUR2A (provided by Dr Andrew Tinker), Kir3.4, Kir2.1, and CHO cell lines stably expressing hERG K_V_1.5, K_V_4.3, Na_V_1.5 were maintained in media containing 10% FCS and appropriate selection antibiotic. Cells were grown either in suspension or in T-flasks and routinely passaged. Cells for patch clamping experiments were plated onto glass cover slips prior to use. Cells for automated patch clamping experiments were freshly prepared on each experimental day.

#### Cloned cardiac ion channel conventional electrophysiology

Standard giga-seal whole-cell patch-clamp techniques were performed at room temperature using glass pipettes (2–5 MΩ). HEKA EPC9/10 amplifiers and Pulse software were used for data acquisition. Series resistance was compensated by >70%. Experimental solutions are given under Supporting information. Voltage protocols were: Kir3.1/3.4, Kir3.4/3.4 Kir2.1 (V_Hold_ −60 mV, +60 mV/100 ms, ramp +140 mV/500 ms, −140 mV/100 ms, 0.1 Hz), hERG (V_Hold_ −80 mV, −40 mV/50 ms, +40 mV/1 s, −40 mV/5 s, 0.1 Hz), K_V_1.5 (V_Hold_ −80 mV, 0 mV/900 ms, −40 mV/100 ms, 0.1 Hz), K_V_4.3 (V_Hold_ −80 mV, +30 mV/0.5 s, 0.067 Hz), Na_V_1.5 (V_Hold_ −100 or −70 mV, −10 mV/20 ms, 1 or 5 Hz) and Kir6.2/SUR2A (V_Hold_ −60 mV, −50 mV/200 ms, ramp −70 mV/250 ms, −70 mV/200 ms, 0.1 Hz), K_V_7.1/KCNE1 (V_Hold_ −80 mV, −50 mV/30 ms, +60 mV/4 s, −50 mV/4 s, 0.05 Hz).

Ionic current recordings were analyzed using HEKA software. Kir2, Kir3 as mean current at −120 mV, hERG and K_V_7 as peak tail current on repolarization to −40/−50 mV. Na_V_1.5 and K_V_4.3 as peak current on depolarization, K_V_1.5 as mean current at the end of the depolarizing pulse.

#### Assessment of cardiac calcium channel activity

Counter screening against the pore forming subunit of the cardiac L-type Ca^2+^ channel was performed using a 96-well plate-based fluorescence plate. Briefly, HEK293 cells expressing Ca_V_1.2 were seeded at 15,000 cells/well. Cells were loaded with Fluo-4 AM dye before being equilibrated in the presence of either a range of concentrations of compound (1, 3, 10 μM) or positive control (1 & 3 μM nimodipine) or vehicle. Plates were placed on a temperature-controlled (37 °C) fluorescence plate reader with liquid handling capability. Following recording of baseline fluorescence of each well (1 min per column, 0.2 Hz), a high-K^+^ stimulus buffer containing the agonist FPL-64176 (600 nM) was applied a column at a time during which time fluorescence was measured and recorded (3 min). Composition of experimental solutions is given in Tables S7-2 and S8 under Supporting Information.

#### Test substance, positive control, bath and pipette solutions

For electrophysiological studies, a 10 mM stock solution of BP-G1 was formulated in DMSO and frozen and stored as aliquots at approximately −20 °C until use. Serial dilution of the 10 mM stock in DMSO was performed in glass vials prior to dilution in external bath solution to achieve the desired final perfusion concentration with a vehicle concentration of 0.1 to 0.3% DMSO. Compound containing experimental solutions was made fresh throughout the experimental day in glass vials. All drug-containing solutions were made up in glassware. Drug delivery systems were constructed from PTFE and glass to minimize drug absorption and adsorption.

#### *In vivo* electrophysiological testing and administration of BP-G1

Mice were anesthetized (1.5% isofluorane), and a 1.2 French octapolar catheter (Millar) with a drug delivery port was placed transvenously, through the jugular vein, into the right atrium. Continuous ECG was recorded using the Animal Bio Amp and PowerLab 4/35 Advanced Instruments apparatus. Acute BP-G1 or the inactive form of BP-G1(SR) administration was achieved by injection through the jugular vein (40 μl of 240 μM). Saline control at the same volume was performed. Results are averages ±SD.

### Radioligand binding assessment of pharmacology

The pharmacology of BP-G1 was further investigated in a number of commercially available radioligand binding assays to determine specific binding to a diverse panel of 80 receptors, GPRCs, ion channels, and transporters. Details of the radioligand binding assay including radioligands, cold ligand, conditions, and the reference compound run for each assay are summarized under Supporting information. In assays where specific binding was observed to be >50% at a single concentration of 10 μM, assays were rerun using eight concentrations (30 nM–100 μM) and normalized specific binding data were plotted against concentration and fitted with a sigmoidal function to determine IC_50_, n_H_ and K_i_. In assays where specific binding was <50% at 10 μM, the IC_50_ and K_i_ were assumed to be >10 μM.

### Molecular dynamics simulations for BP-G1-Kir3.1/4 channel interactions

The predicted BP-G1-Kir3.1/4 channel complex was immersed in an explicit lipid bilayer of POPC, POPE, POPS, and cholesterol with molecular ratio of 25:5:5:1 (37) and a water box in 107.4 Å × 107.4 Å × 154.5 Å dimension by using the CHARMM-GUI Membrane Builder webserver (http://www.charmm-gui.org/?doc=input/membrane). Four PIP_2_ molecules, 150 mM KCl, 2 K^+^, 2 water molecules (located in the selectivity filter as obtained from the crystal structures), and extra neutralizing counter ions Cl^−^ were added into the system. The total atoms of the system were 164,906 (Fig. S2). The Antechamber module of AmberTools was used to generate the parameters for PIP_2_ and BP-G1 using the general AMBER force field (GAFF). The partial charges for the BP-G1 were calculated using restrained electrostatic potential (RESP) charge-fitting scheme by *ab initio* quantum chemistry at the HF/6-31G∗ level (GAUSSIAN 09 program) (38, 39). The PMEMD.CUDA program in AMBER16 was used to conduct the MD simulations. The MD simulations were performed with periodic boundary conditions to produce isothermal-isobaric ensembles. Long-range electrostatics were calculated using the particle mesh Ewald (PME) method (40) with a 10 Å cutoff. Prior to production runs, energy minimization of the system was carried out. Subsequently, the system was heated from 0 K to 303 K using Langevin dynamics with the collision frequency of 1 ps^−1^. During the heating, the BP-G1-Channel complex, PIP_2_ was position-restrained using an initial constant force of 500 kcal/mol/Å^2^ and weakened to 10 kcal/mol/Å^2^, allowing lipid and water molecules to move freely. Then, the system went through 5 ns equilibrium MD simulations. Finally, a total of 1μs production MD simulation was conducted, and coordinates were saved every 50 ps for analysis.

### Chemical synthesis

For the experiments presented in this article, some of the BP-G1 and its inactive isomer were obtained through academic and industrial partnerships not disclosed here. Related analog compounds, GAT1572 (compound 4, Fig. 6*A*), GAT1573 (compound 6, Fig. 6*A*), and GAT1588, and intermediates were all synthesized at Northeastern University. BP-G1 was synthesized and confirmed by LCMS; however, final purification proved challenging with a low yielding reaction. Improvement on the BP-G1 synthesis yield will be the focus of future experiments. All reagents were purchased from commercial sources as reagent grade. Reactions were monitored by thin-layer chromatography (TLC) using commercially prepared silica gel 60 F254 glass plates. Compounds were visualized under ultraviolet (UV) light or by staining with iodine. Flash column chromatography was carried out on an autoflash purification unit using prepacked columns from Biotage and Buchi. Solvents used include hexanes and ethyl acetate, methanol, dichloromethane for purification. Characterization of compounds and their purity was established by a combination of LCMS, TLC, and NMR analyses. NMR spectra were recorded in CDCl3 on a NMR spectrometer (1H NMR at 400 MHz). Chemical shifts were recorded in parts per million (δ) relative to tetramethylsilane (TMS; 0.00 ppm) or solvent peaks as the internal reference. Multiplicities are indicated as br (broadened), s (singlet), d (doublet), t (triplet), q (quartet), quin (quintet), and m (multiplet). Coupling constants (J) are reported in hertz (Hz). All test compounds were greater than 95% pure, as determined by LC-MS analysis performed with a dual-wavelength UV-visible detector and quadrupole mass spectrometer.

#### 2,2-dimethyl-6-nitro-2H-chromene (Fig. 6A, compound 3)

The compound was synthesized using nitrophenol (compound 2) (1.0 g, mmol), 1,1-diethoxy-3-methylbut-2-ene (compound 1) (1 g, 7.19 mmol), pyridine (142 mg, 1.79 mmol) and loading in a 100 ml round bottom flask. Starting material was dissolved in anhydrous toluene and refluxed for 12 h. The reaction mixture was poured into water and extracted with ethyl acetate (3 × 50 ml). The combined organic layers were washed with water (20 ml) and brine (20 ml), dried over Na_2_SO_4_, and evaporated under vacuum. The crude residue was purified by silica gel column chromatography (EtOAc/hexane) with the compound eluting in 100% hexane to yield 700 mg, of light yellow oil: 1H NMR (400 MHz, CDCl3): δ 8.01(d, J = 11.5 Hz, 1H), 7.89 (s, 1H), 6.81 (d, J = 9.0 Hz, 1H), 6.35 (d, J = 12.0 Hz, 1H), 5.75 (d, J = 13.0 Hz, 1H), 1.48 (s, 6H).

#### 2,2,9-trimethyl-2H-pyrano[2,3-g]quinoline (4; GAT1572)

i) The compound was synthesized starting with the reduction of compound (Fig. 6*A*, compound 3) (700 mg, mmol) to 3 2,2-dimethyl-2H-chromen-6-amine by using Tin (II) Chloride (3.2 g, 17.07 mmol), dissolved in ethanol, and refluxed for 12 h. Upon completion the reaction was then concentrated to dry to remove the ethanol and then dissolved in ethyl acetate. The reaction was then quenched with aqueous sodium bicarbonate solution and then filtered through silica to remove the metal tin from the mixture. The layers were then separated after filtering and dried over sodium sulfate and concentrated to dry. This compound was taken ahead without further purification: ii) compound was synthesized by dissolving 2,2-dimethyl-2H-chromen-6-amine crude (300 mg), Ferric Chloride coated silica (300 mg) were dissolved in acetic acid and stirred at room temperature, and methyl vinyl ketone was added dropwise (0.2 ml) and stirred at 70 °C for 1 h. ii) Next, Zinc (II) Chloride (300 mg) and Indium (III) Chloride (50 mg, mmol) were added to the reaction mixture and refluxed for 2 h under argon. The reaction was then concentrated to dry to remove acetic acid, then redissolved in ethyl acetate and quenched with aqueous and solid sodium sulfate until effervescence stopped. The reaction mixture was then separated, and the organic layer was dried over sodium sulfate, Na_2_SO_4_ and evaporated under vacuum. The crude residue was purified by silica gel column chromatography (EtOAc/hexane) to yield the desired 120 mg, of a light brown oily solid: 1H NMR (400 MHz, CDCl3 δ 8.57(d, J = 5.0 Hz, 1H), 7.67 (s, 1H), 7.24 (s, 1H), 7.11 (d, J = 4.5 Hz, 1H), 6.58 (d, J = 10.0 Hz, 1H), 5.90 (d, J = 10.0 Hz, 1H), 2.59(s, 3H), 1.50 (s, 6H). LC/MS: m/z calculated for C15H15NO [M + H]+, 226.29.

#### (3R,4S)-2,2,9-trimethyl-4-(phenethylamino)-3,4-dihydro-2H-pyrano[2,3-g]quinolin-3-ol (GAT1588)

GAT1572 (compound 4) (200 mg) was dissolved in ethyl acetate and 1-methylimidazole (0.5 ml) and R-R Mn Salen catalyst (120 mg). Aqueous sodium hypochlorite (2 ml) was then added dropwise to the reaction and stirred at RT for 24 h to yield the epoxide intermediate (compound 5). The reaction mixture was poured into water and extracted with ethyl acetate (3 × 50 ml). The combined organic layers were washed with water (20 ml) and brine (20 ml), dried over Na_2_SO_4_, and evaporated under vacuum. The epoxide crude was taken ahead without further purification. The Compound 5 (100 mg, mmol) was dissolved in 1 to 4 dioxane followed by the addition of lithium perchlorate (170 mg, mmol), 2-pheneythylamine (2 ml), and refluxed for 12 h to yield mg, % yield, of a solid: 1H NMR (400 MHz, CDCl3) δ 8.57(d, J = 5.0 Hz, 1H), 7.67 (s, 1H), 7.24 (s, 1H), 7.11 (d, J = 4.5 Hz, 1H), 6.58 (d, J = 10.0 Hz, 1H), 5.90 (d, J = 10.0 Hz, 1H), 2.59 (s, 3H), 1.50 (s, 6H). LC/MS: m/z calculated for C23H26N2O2 [M + H]+, 363.47.

#### (2,2,9-trimethyl-2H-pyrano[2,3-g]quinolin-7-yl)methanol (compound 6; GAT1573)

Compound 5 (100 mg, mmol), para toluene sulfonic acid (150 mg,), Na_2_S_2_O_8_ (400 mg) were added to a screwcap vial and dissolved in methanol:water (7:3) and heated to 130 °C and stirred for 12 h, to yield mg, % yield, of a solid: 1H NMR (400 MHz, CDCl3 δ 7.65 (s, 1H), 7.24 (s, 1H), 7.00 s, 1H), 6.57 (d, J = 10.0 Hz, 1H), 5.90 (d, J = 10.0 Hz, 1H), 4.80 (s, 2H), 2.59 (s, 3H), 1.57 (s, 1H), 1.50 (s, 6H). LC/MS: m/z calculated for C16H17NO2 [M + H]+, 256.32.

**Figure:**
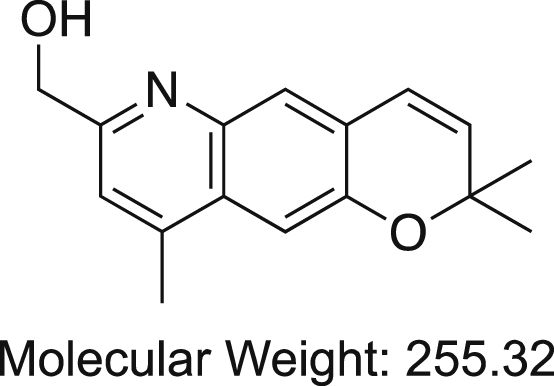

## Data availability

All data are contained within the article.

## Supporting information

This article contains supporting information.

## Conflicts of interest

The authors declare that they have no conflicts of interest with the contents of this article.

## Supporting information

Supplemental Figures S1–S5, Tables S1–S8 and Equations S1–S2
