# Supplementary material for: A benzopyran with antiarrhythmic activity is an inhibitor of Kir3.1-containing potassium channels
Source: J Biol Chem. 2021 Mar 11;296:100535. doi: 10.1016/j.jbc.2021.100535 (PMC8086025; doi:10.1016/j.jbc.2021.100535)
Supplement: Supplemental Figures S1–S5, Tables S1–S8 and Equations S1–S2 [file mmc1.pdf]

A benzopyran with anti-arrhythmic activity is an inhibitor of Kir3.1-containing potassium channels

Supplementary materials

Meng Cui[1]*, Yaser Alhamshari[1], Lucas Cantwell[1], Said El-Haou[3], Giasemi C. Eptaminitaki[1], Mengmeng Chang[2], Obada Abou-Assali[2], Haozhou Tan[1], Keman Xu[1], Meghan Masotti[1], Leigh D. Plant[1,5], Ganesh A. Thakur[1], Sami F. Noujaim[2], James Milnes[3], and Diomedes E. Logothetis[1,4,5]*

[1] Department of Pharmaceutical Sciences, School of Pharmacy, Bouvé College of Health Sciences, Northeastern University, Boston, Massachusetts, 02115, USA

[2] Department of Molecular Pharmacology & Physiology, University of South Florida, Morsani College of Medicine, Tampa, FL, 33612, USA

[3] Department of Cardiac Biology, Xention Ltd, Cambridge, CB22 3EG, UK

[4] Department of Chemistry and Chemical Biology, College of Science, Northeastern University, Boston, MA 02115, USA

[5] Center for Drug Discovery, Northeastern University, Boston, MA 02115, USA

* To whom correspondence should be addressed: m.cui@northeastern.edu (MC); d.logothetis@northeastern.edu (DEL)

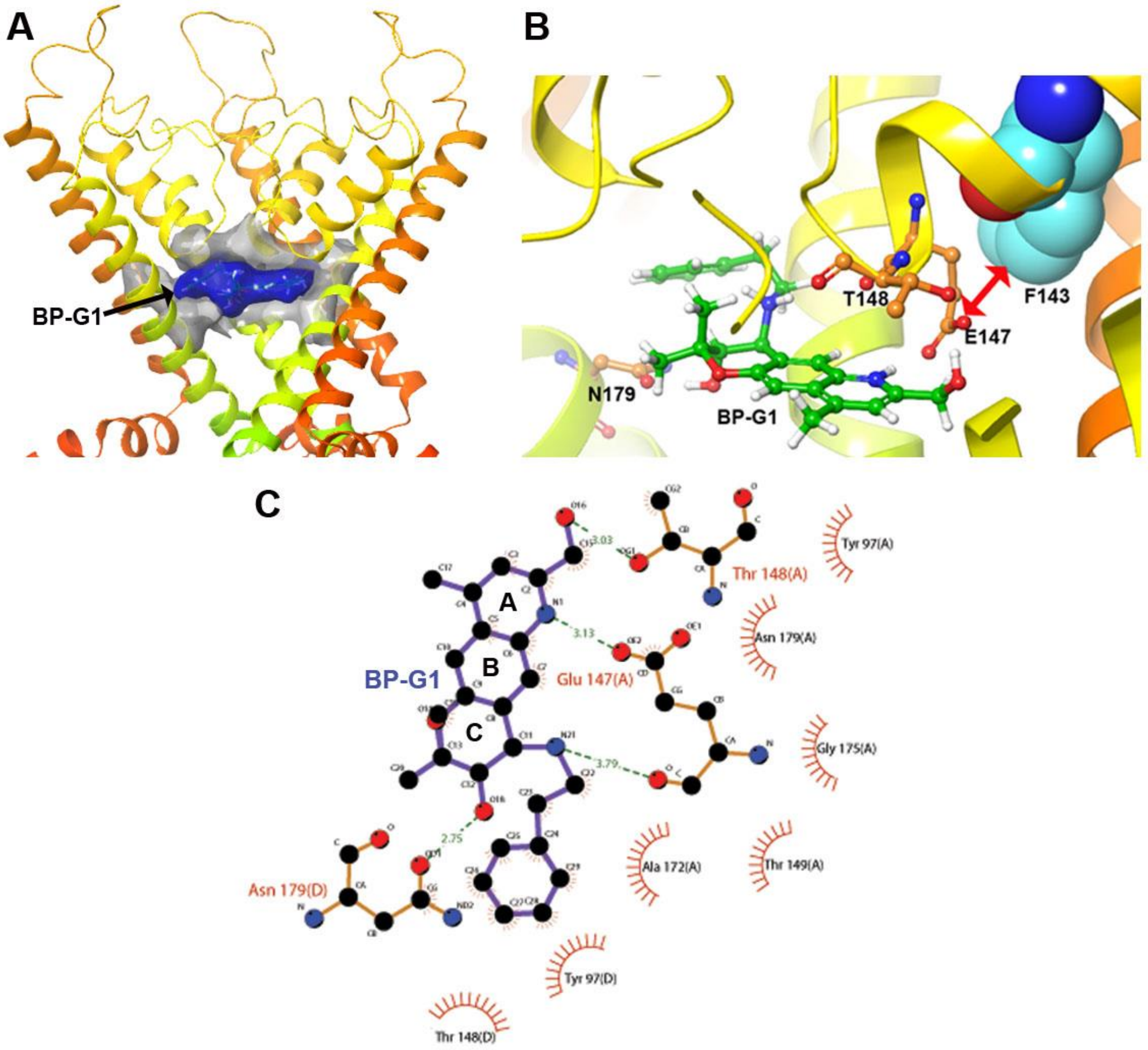

Fig. S1. Docking and predicted interactions of BP-G1 with a Kir3.4 model. (A) Predicted binding pocket in the Kir3.4/4(S143F) channel for BP-G1. (B) Molecular model of Kir3.4/4(S143F) channel binding site with the docked BP-G1. The channel model is shown in Ribbons presentation. The BP-G1 and hydrogen-bonding interaction residues are drawn in ball and stick, and residue F143 is shown in CPK sphere. The red arrow shows the residue E147 is repositioned by residue F143 in the Kir3.4 (S143F) channel mutant to interact with the BP-G1 compound through one hydrogen bond and one salt bridge. (C) The schematic depiction of the main interactions (in two dimensions) is shown for Kir3.4/4(S143F) with the BP-G1 compound (by LIGPLOT software).

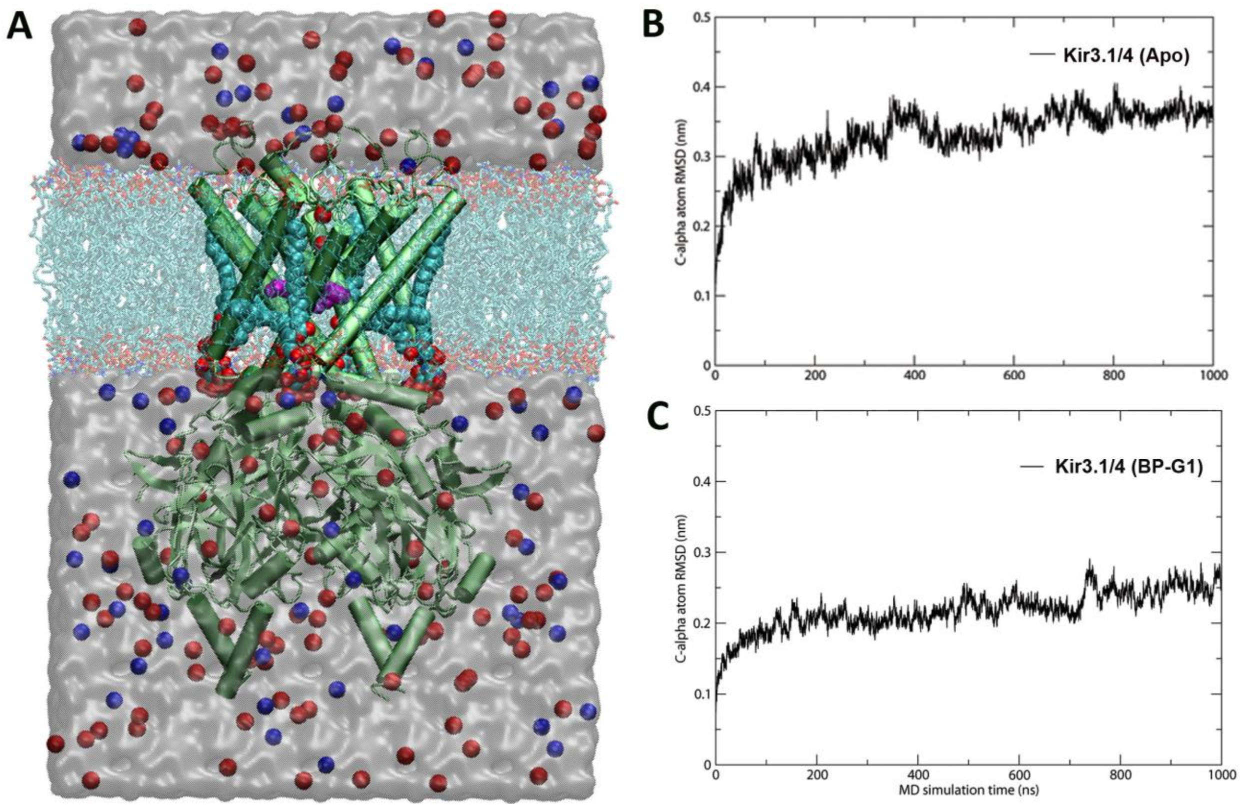

Fig. S2. (A) The BP-G1-Kir3.1/4 complex in lipid bilayer/water environment for MD simulations. The Kir3.1/4 is drawn in Cartoon (green), the BP-G1 in VDW (magenta), PIP_2 in VDW (colored by atom names), lipids, POPC, POPE, POPS, and cholesterol in Licorice (colored by atom names), water in transparent surface (grey), potassium ions (red), chloride ions (blue). RMSD of C-alpha atoms of Kir3.1/4 channel (B) and Kir3.1/4-BP-G1 (C).

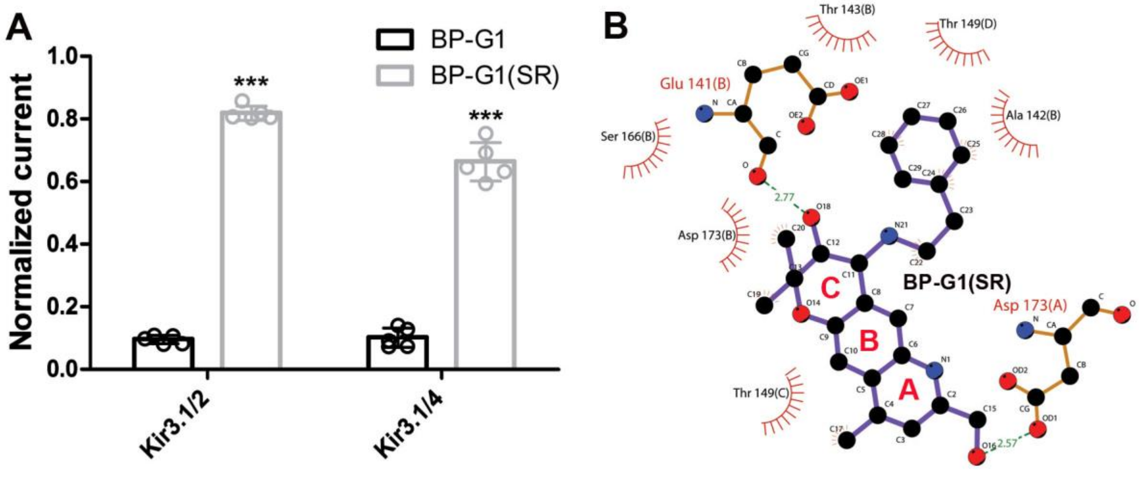

Fig. S3. (A) The BP-G1(SR) enantiomer nominally inhibits Kir3.1/2 and Kir3.1/4 current. (B) BP-G1(SR) forms two hydrogen bonds with Kir3.1/4 channel (Ligplot). The asterisks indicate significant differences tested by unpaired Student's t test (***$p < 0.001$) compared to BP-G1, (data are mean ± SD, N=5).

Table S1. Comparison of inhibitory activity of published NTC-801 data with Benzopyran-G1 compound on different cardiac ion channels, (MPC: manual patch clamp). Table S1 values for BP-G1 were determined by using the equations S1 and S2 shown in Appendix 4

Ion Channel	NTC-801 Published IC_{50}	BP-G1
Kir3.1/3.4	0.7nM ^{oocyte}	10.5 nM
Kir3.4	ND	19.8 µM
Kir3.1/3.2	34 nM	32.2 nM
GP I_{KACh}	7nM	No data
hERG	>30µM	>5 µM
Na_v1.5 (1Hz)	8.3µM	>>10 µM ^{MPC}
NaV1.2		>>10µM
Ca_v1.2	$I_{Ca,L}$ >30µM	No data
K_{ir}2.1	>30µM	>>10µM ^{MPC}
K_{ir}6.2/SUR2A	I_{KATP} 7.8µM	>10µM
Kir6.1/SUR2B		9.9µM
Kv1.5	>30µM	16.3µM ^{MPC}
Kv4.3	>11.6µM	11.3 µM
HCN4	>30µM	No data
Kv7.1/KCNE1	>30µM	No data
Kv1.7		7.0µM

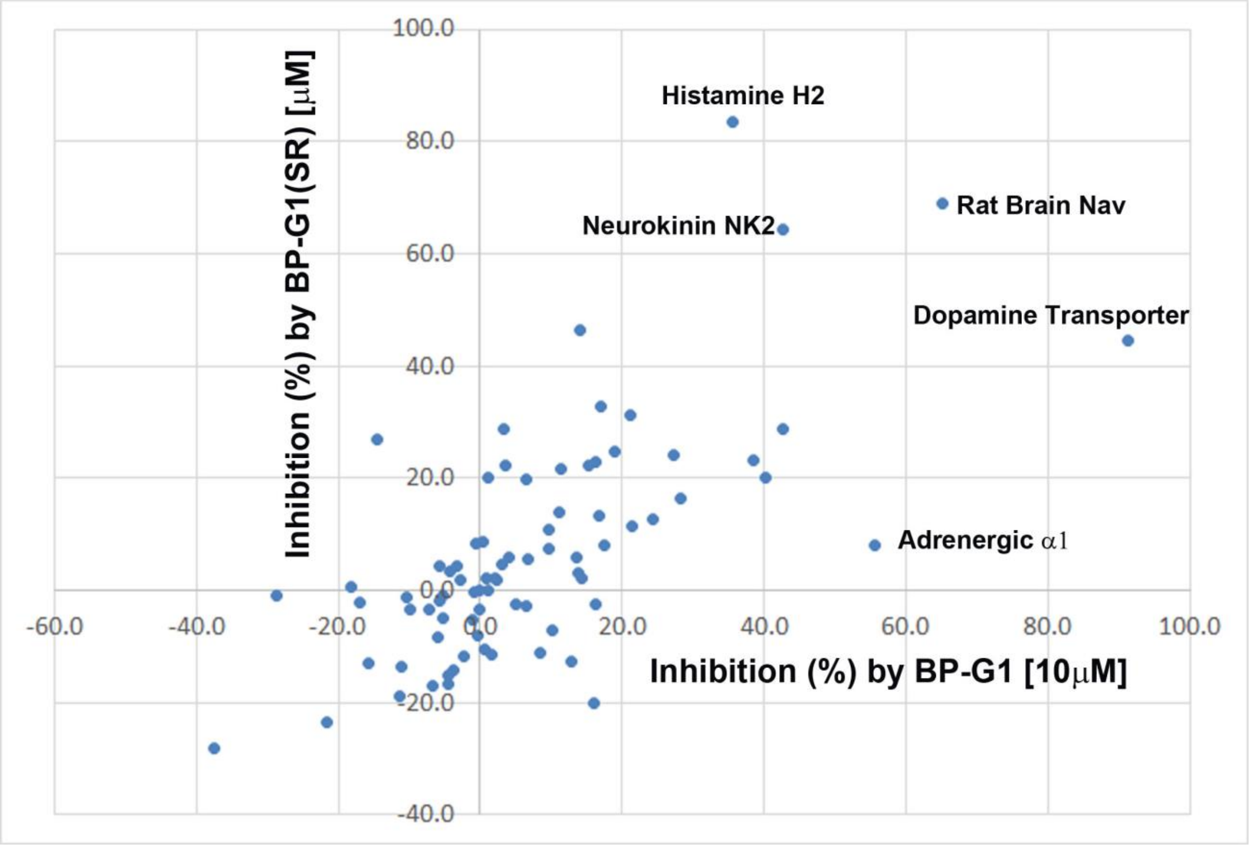

Figure S4. Radioligand Binding – Inhibition Data at 10µM. In vitro binding assays were used to investigate the potential to interact with a diverse panel of 80 nuclear receptors, GPCRs, ion channels, ligand-gated channels, transporters, kinases. Any potential interactions (>50% specific binding at 10 µM) were followed up with full binding curves and/or functional assays. If less than 50% specific binding was observed at 10µM, no further investigation of these targets was pursued.

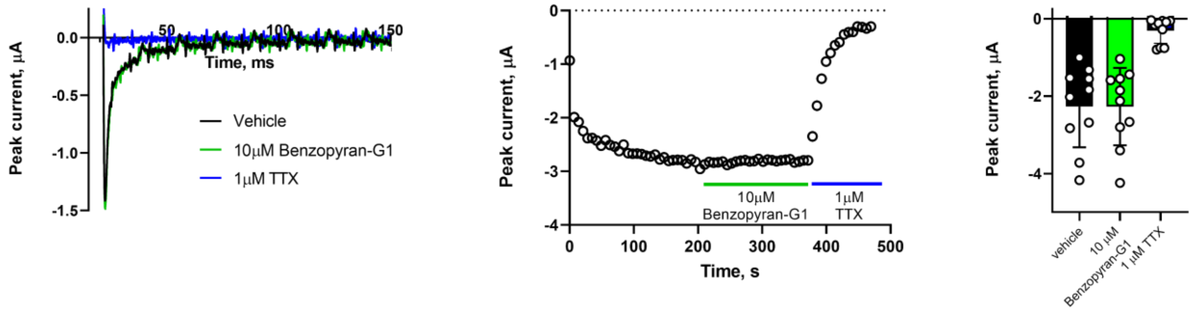

Fig. S5. (A) Overlay of sample traces from a representative experiment showing currents evoked when oocytes expressing rat $Na_V1.2$ and $Na_V\beta1$ are pulsed from -90 mV to -10 mV in the presence of the vehicle (96mM Na^+; black), 10 µM BP-G1 (green) or 1µM of the channel pore blocker, TTX (blue). (B) Experimental time course showing the effect of 10 µM BP-G1 or 1 µM TTX perfusion on $Na_V1.2$ currents. (C) Average currents were -2.16 ± 0.36 µA with the vehicle; -2.17µA ± 0.36 µA with10 µM BP-G1, and -0.31 ± 0.32 µA with 1 µM TTX . Data are mean ± SD. Significance was tested by unpaired Student's t test (N=10).

Table S2. Inhibitory activity of Benzopyran-G1 on protein targets

	Source	Ligand	Ligand concentration	Kd (nM)	Non specific	Incubation	Detection method	Reference:	Specific Binding 10 µM	% Specific Binding at 10 µM
Adenosine A1	human recombinant (CHO cells)	[3H]DPCPX	1 nM	1.7 nM	DPCPX (1 µM)	60 min/RT	Scintillation counting	DPCPX (IC 50:1 nM)	38.400	<50% @10 µM
Adenosine A2A	human recombinant (HEK-293 cells)	[3H]CGS 21680	6 nM	27 nM	NECA (10 µM)	120 min/RT	Scintillation counting	NECA (IC5 0:28 nM)	-28.800	<50% @10 µM
Adenosine A3	human recombinant (HEK-293 cells)	[125I]AB-MECA	0.15 nM	0.22 nM	IB-MECA (1 µM)	120 min/RT	Scintillation counting	IB-MECA (IC5 0:0.27 nM)	18.900	<50% @10 µM
Adrenergic α1	rat cerebral cortex	[3H]prazosin	0.25 nM	0.09 nM	prazosin (0.5 µM)	60 min/RT	Scintillation counting	prazosin (IC 50:0.182 n M	55.567	
Adrenergic α2	rat cerebral cortex	[3H]prazosin	0.25 nM	0.09 nM	prazosin (0.5 µM)	60 min/RT	Scintillation counting	prazosin (IC 50:0.182 n M	0.300	<50% @10 µM
Adrenergic β1	rat cerebral cortex	[3H]RX 821002	0.5 nM	0.38 nM	(-)epinephrine (100 µM)	60 min/RT	Scintillation counting	yohimbine (IC50:58.7 n M)	1.233	<50% @10 µM
Adrenergic β 2	human recombinant (HEK-293 cells)	[3H](-)CGP 12177	0.3 nM	0.39 nM	alprenolol (50 µM)	60 min/RT	Scintillation counting	atenolol (IC 50:278 nM)	3.033	<50% @10 µM
Angiotensin-II AT1	human recombinant (HEK-293 cells)	"[125I][Sar1,Ile8]-AT-II"	0.05 nM	0.05 nM	angiotensin-II (10 µM)	120 min/37°C	Scintillation counting	saralasin (I C50:0.63 n M)	-10.267	<50% @10 µM
Angiotensin-II AT2	human recombinant (HEK-293 cells)	[125I]CGP 42112A	0.01 nM	0.01 nM	angiotensin-II (1 µM)	4 hr/37°C	Scintillation counting	angiotensin-II (IC50:0.1 4 nM	-0.100	<50% @10 µM
Bombesin BB	rat cerebral cortex	[125I][Tyr4]bombesin	0.01 nM	0.71 nM	bombesin (1 µM)	60 min/RT	Scintillation counting	bombesin (I C50:0.205 nM)	-6.600	<50% @10 µM
Benzodiazepine BDZ Peripheral	rat heart	[3H]PK 11195	0.2 nM	1.8 nM	PK 11195 (10 µM)	15 min/RT	Scintillation counting	PK 11195 (IC5 0:0.98 nM)	-14.433	<50% @10 µM
Benzodiazepine BDZ Central	rat cerebral cortex	[3H]flunitrazepam	0.4 nM	2.1 nM	diazepam (3 µM)	60 min/4°C	Scintillation counting	diazepam (I C50:8.6 nM)	12.933	<50% @10 µM
Bradykinin B2	human recombinant (CHO cells)	[3H]desArg10-KD	0.35 nM	0.085 nM	desArg9[Leu8]-BK (10 µM)	60 min/RT	Scintillation counting	desArg10-KD (IC50:0. 77 nM)	-7.2	<50% @10 µM
Calcitonin gene-related peptide CGRP	human recombinant (CHO cells)	[125I]hCGRPa	0.03 nM	0.06 nM	hCGRPa (1 µM)	90 min/RT	Scintillation counting	hCGRPa (I C50:0.058 nM)	-15.800	<50% @10 µM
Canabinoid CB1	human recombinant (CHO cells)	[3H]CP 55940	0.5 nM	3.5 nM	WIN 55212-2 (10 µM)	120 min/37°C	Scintillation counting	CP 55940 (IC5 0:0.58 nM)	-1.167	<50% @10 µM
"Cholecystokinin CCK1 (CCKA), "	human recombinant (CHO cells)	[125I]CCK-8s	0.08 nM	0.24 nM	CCK-8s (1 µM)	60 min/RT	Scintillation counting	CCK-8s (IC50:0. 1 nM)	-16.967	<50% @10 µM
Cholecystokinin CCK2 (CCKB)	human recombinant (CHO cells)	[125I]CCK-8s	0.08 nM	0.054 nM	CCK-8s (1 µM)	60 min/RT	Scintillation counting	CCK-8s (IC50:0. 098 nM	-18.233	<50% @10 µM
Dopamine D1	human recombinant (CHO cells)	[3H]SCH 23390	0.3 nM	0.2 nM	SCH 23390 (1 µM)	60 min/RT	Scintillation counting	SCH 23390 (IC5 0:0.242 nM)	21.300	<50% @10 µM
Dopamine D2S	human recombinant (HEK-293 cells)	[3H]methyl-spiperone	0.3 nM	0.15 nM	(+)butaclamol (10 µM)	60 min/RT	Scintillation counting	(+)butaclamol (IC50:1.6 4 nM)	-4.300	<50% @10 µM
Dopamine D3	human recombinant (CHO cells)	[3H]methyl-spiperone	0.3 nM	0.085 nM	(+)butaclamol (10 µM)	60 min/RT	Scintillation counting	(+)butaclamol (IC50:1.4 nM)	16.167	<50% @10 µM
Dopamine D4.4	human recombinant (CHO cells)	[3H]methyl-spiperone	0.3 nM	0.19 nM	(+)butaclamol (10 µM)	60 min/RT	Scintillation counting	clozapine (I C50:46.5 n M	5.133	<50% @10 µM
Dopamine D5	human recombinant (GH4 cells)	[3H]SCH 23390	0.3 nM	0.25 nM	SCH 23390 (10 µM)	60 min/RT	Scintillation counting	SCH 23390 (IC5 0:0.39 nM)	6.400	<50% @10 µM

Endothelin ETA	human recombinant (CHO cells)	[125I]endothelin-1	0.03 nM	0.03 nM	endothelin-1 (100 nM)	120 min/37°C	Scintillation counting	endothelin-1 (IC50:0.038 nM	9.600	<50% @10 µM
Endothelin ETB	human recombinant (CHO cells)	[125I]endothelin-1	0.03 nM	0.04 nM	endothelin-1 (0.1 µM)	120 min/37°C	Scintillation counting	endothelin-3 (IC50:0.03 nM)	-11.100	<50% @10 µM
GABA (non-selective)	rat cerebral cortex	[3H]GABA	10 nM	15 nM	GABA (100 µM)	60 min/RT	Scintillation counting	GABA (IC50:38 nM)	-0.733	<50% @10 µM
GAL1	human recombinant (HEK-293 cells)	[125I]galanin	0.1 nM	0.1 nM	galanin (1 µM)	60 min/RT	Scintillation counting	galanin (IC50:0.35 nM	-0.100	<50% @10 µM
GAL2	human recombinant (CHO cells)	[125I]galanin	0.05 nM	0.63 nM	galanin (1 µM)	120 min/RT	Scintillation counting	galanin (IC50:0.3 nM	-0.333	<50% @10 µM
PDGF	Balb/c 3T3 cells	[125I]PDGF BB	0.03 nM	0.15 nM	PDGF BB (10 nM)	180 min/4°C	Scintillation counting	PDGF BB (IC50:0.093 nM)	-21.533	<50% @10 µM
Chemokines CCR1	human recombinant (HEK-293 cells)	[125I]MIP-1a	0.01 nM	0.02 nM	MIP-1a (100 nM)	120 min/RT	Scintillation counting	MIP-1a (IC50:0.04 nM)	8.433	<50% @10 µM
Chemokines CXCR2 (IL-8B)	human recombinant (HEK-293 cells)	[125I]IL-8	0.025 nM	0.022 nM	IL-8 (30 nM)	60 min/RT	Scintillation counting	IL-8 (IC50:0.115 nM)	2.300	<50% @10 µM
Histamine H1	human recombinant (HEK-293 cells)	[3H]pyrilamine	1 nM	1.7 nM	pyrilamine (1 µM)	60 min/RT	Scintillation counting	pyrilamine (IC50:2.2 nM	-5.300	<50% @10 µM
Histamine H2	human recombinant (CHO cells)	[125I]APT	0.075 nM	2.9 nM	tiotidine (100 µM)	120 min/RT	Scintillation counting	cimetidine (IC50:350 nM)	35.533	<50% @10 µM
Melanocortin MC4	human recombinant (CHO cells)	[125I]NDP-a-MSH	0.05 nM	0.54 nM	NDP-a-MSH (1 µM)	120 min/37°C	Scintillation counting	NDP-a-MSH (IC50:0.23 nM)	6.800	<50% @10 µM
Melatonin MT1	human recombinant (CHO cells)	[125I]2-iodomelatonin	0.01 nM	0.04 nM	melatonin (1 µM)	60 min/RT	Scintillation counting	melatonin (IC50:0.3 nM)	16.233	<50% @10 µM
Muscarinic M1	human recombinant (CHO cells)	[3H]pirenzepine	2 nM	13 nM	atropine (1 µM)	60 min/RT	Scintillation counting	pirenzepine (IC50:22 nM)	-5.600	<50% @10 µM
Muscarinic M2	human recombinant (CHO cells)	[3H]AF-DX 384	2 nM	4.6 nM	atropine (1 µM)	60 min/RT	Scintillation counting	methoctramine (IC50:32 nM)	-5.700	<50% @10 µM
Muscarinic M3	human recombinant (CHO cells)	[3H]AF-DX 384	2 nM	4.6 nM	atropine (1 µM)	60 min/RT	Scintillation counting	methoctramine (IC50:32 nM)	1.033	<50% @10 µM
Muscarinic M4	human recombinant (CHO cells)	[3H]4-DAMP	0.2 nM	0.32 nM	atropine (1 µM)	60 min/RT	Scintillation counting	4-DAMP (IC50:0.53 nM)	11.100	<50% @10 µM
Muscarinic M5	human recombinant (CHO cells)	[3H]4-DAMP	0.3 nM	0.3 nM	atropine (1 µM)	60 min/RT	Scintillation counting	4-DAMP (IC50:0.52 nM	-0.567	<50% @10 µM
Neurokinin NK1	U373MG uppsala	[125I]-Substance P LYS3	0.05 nM	0.04 nM	"[Sar9,Met(O2)11]-SP (1 µM)"	30 min/RT	Scintillation counting	"[Sar9,Met(O2)11]-SP (IC50:0.29 nM)"	42.633	<50% @10 µM
Neurokinin NK2	human recombinant (CHO cells)	[125I]NKA	0.1 nM	0.12 nM	[Nleu10]-NKA (4-10) (300 nM)	60 min/RT	Scintillation counting	[Nleu10]-NKA (4-10) (IC50:2.7 nM)	3.500	<50% @10 µM
Neurokinin NK3	human recombinant (CHO cells)	[3H]SR 142801	0.4 nM	0.47 nM	SB 222200 (10 µM)	120 min/RT	Scintillation counting	SB 222200 (IC50:8.8 nM)	2.033	<50% @10 µM
Neuropeptide-Y Y1	SK-N-MC cells (endogenous)	[125I]peptide YY	0.025 nM	0.06 nM	NPY (1 µM)	120 min/37°C	Scintillation counting	NPY (IC50:0.079 nM)	-2.300	<50% @10 µM
Neuropeptide-Y Y2	KAN-TS cells	[125I]peptide YY	0.015 nM	0.01 nM	NPY (1 µM)	60 min/37°C	Scintillation counting	NPY (IC50:0.0555 nM)	0.733	<50% @10 µM
Neurotensin NTS1 (NT1)	human recombinant (CHO cells)	[125I]Tyr3-neurotensin	0.05 nM	0.22 nM	neurotensin (1 µM)	60 min/4°C	Scintillation counting	neurotensin (IC50:0.3 nM)	-4.467	<50% @10 µM
Opioid δ2	human recombinant (CHO cells)	[3H]DADLE	0.5 nM	0.73 nM	naltrexone (10 µM)	120 min/RT	Scintillation counting	DPDPE (IC50:1.8 nM)	-2.767	<50% @10 µM
Opioid Kappa	rat recombinant (CHO cells)	[3H]U 69593	1 nM	2 nM	naloxone (10 µM)	60 min/RT	Scintillation counting	U 50488 (IC50:0.84 nM)	21.267	<50% @10 µM

Opioid µ	human recombinant (HEK-293 cells)	[3H]DAMGO	0.5 nM	0.35 nM	naloxone (10 µM)	120 min/RT	Scintillation counting	DAMGO (IC50:0.537 nM	24.333	<50% @10 µM
NOP (ORL1)	human recombinant (HEK-293 cells)	[3H]nociceptin	0.2 nM	0.4 nM	nociceptin (1 µM)	60 min/RT	Scintillation counting	nociceptin (IC50:0.5 nM)	16.833	<50% @10 µM
PCP	rat cerebral cortex	[3H]TCP	10 nM	13 nM	MK 801 (10 µM)	120 min/37°C	Scintillation counting	MK 801 (IC50:5.3 nM)	1.700	<50% @10 µM
EP2	human recombinant (HEK-293 cells)	[3H]PGE2	3 nM	3 nM	PGE2 (10 µM)	120 min/RT	Scintillation counting	PGE2 (IC50:1.7 nM)	15.300	<50% @10 µM
EP4	human recombinant (HEK-293 cells)	[3H]PGE2	0.5 nM	0.3 nM	PGE2 (10 µM)	120 min/RT	Scintillation counting	PGE2 (IC50:0.38 nM)	9.667	<50% @10 µM
Prosanoid IP (PGI2)	human recombinant (HEK-293 cells)	[3H]iloprost	6 nM	8 nM	iloprost (10 µM)	60 min/RT	Scintillation counting	iloprost (IC50:22 nM)	11.400	<50% @10 µM
5-HT1A	human recombinant (HEK-293 cells)	[3H]8-OH-DPAT	0.3 nM	0.5 nM	8-OH-DPAT (10 µM)	60 min/RT	Scintillation counting	8-OH-DPAT (IC50:0.36 nM)	27.333	<50% @10 µM
5-HT1B	rat cerebral cortex	[125I]CYP (+ 30 µM isoproterenol)	0.1 nM	0.16 nM	serotonin (10 µM)	120 min/37°C	Scintillation counting	serotonin (IC50:10 nM)	-3.700	<50% @10 µM
5-HT2A	human recombinant (HEK-293 cells)	[3H]ketanserin	0.5 nM	0.6 nM	ketanserin (1 µM)	60 min/RT	Scintillation counting	ketanserin (IC50:0.73 nM	14.100	<50% @10 µM
5-HT2B	human recombinant (CHO cells)	[125I](±)DOI	0.2 nM	0.2 nM	(±)DOI (1 µM)	60 min/RT	Scintillation counting	(±)DOI (IC50:5 nM	16.033	<50% @10 µM
5-HT2C	human recombinant (HEK-293 cells)	[3H]mesulergine	1 nM	0.5 nM	RS 102221(10 µM)	120 min/37°C	Scintillation counting	RS 102221 (IC50:3.1 nM)	-3.200	<50% @10 µM
5-HT3	human recombinant (CHO cells)	[3H]BRL 43694	0.5 nM	1.15 nM	MDL 72222 (10 µM)	120 min/RT	Scintillation counting	MDL 72222 (10 nm)	10.200	<50% @10 µM
5-HT5a	human recombinant (HEK-293 cells)	[3H]LSD	1.5 nM	1.5 nM	serotonin (100 µM)	120 min/37°C	Scintillation counting	serotonin (IC50:120 nM)	28.333	<50% @10 µM
5-HT6	human recombinant (CHO cells)	[3H]LSD	2 nM	1.8 nM	serotonin (100 µM)	120 min/37°C	Scintillation counting	serotonin (IC50:150 nM)	3.200	<50% @10 µM
5-HT7	human recombinant (CHO cells)	[3H]LSD	4 nM	2.3 nM	serotonin (10 µM)	120 min/RT	Scintillation counting	serotonin (IC50:0.45 nM	6.400	<50% @10 µM
Somatostatin sst	AtT-20 cells	[125I]Tyr11-somatostatin-14	0.05 nM	0.08 nM	somatostatin-14 (300 nM)	60 min/37°C	Scintillation counting	somatostatin-14 (IC50:0.2 nM)	-9.933333333	<50% @10 µM
Vasoactive intestinal peptide PAC1 (PACAP)	human recombinant (CHO cells)	[125I]PACAP1-27	0.015 nM	0.092 nM	PACAP1-27 (100 nM)	120 min/RT	Scintillation counting	PACAP1-38 (IC50:0.08 nM)	-37.56666667	<50% @10 µM
Vasoactive intestinal peptide VPAC1 (VIP1)	human recombinant (CHO cells)	[125I]VIP	0.04 nM	0.05 nM	VIP (1 µM)	60 min/RT	Scintillation counting	VIP (IC50:0.28 nM)	-5.933333333	<50% @10 µM
Vasopressin (V1a)	human recombinant (CHO cells)	[3H]AVP	0.3 nM	0.5 nM	AVP (1 µM)	60 min/RT	Scintillation counting	"[d(CH2)51, Tyr(Me)2]-AVP (IC50: 0.89 nM)"	4	<50% @10 µM
Steroid nuclear receptors GR	IM-9 cells (cytosol)	[3H]dexamethasone	1.5 nM	1.5 nM	triamcinolone (10 µM)	6 hr/4°C	Scintillation counting	dexamethasone (IC50:4.3 nM)	13.933	<50% @10 µM
Non-steroid nuclear receptors PPARγ	human recombinant (E. coli)	[3H]rosiglitazone	5 nM	5.7 nM	rosiglitazone (10 µM)	120 min/4°C	Scintillation counting	rosiglitazone (IC50:11 nM)		<50% @10 µM
TNF- α	U-937 cells	[125I]TNF-a	0.1 nM	0.05 nM	TNF-a (10 nM)	120 min/4°C	Scintillation counting	TNF-a (IC50:0.1 nM)	0.967	<50% @10 µM

sigma (non-selective)	Jurkat cells (endogenous)	[3H]DTG	10 nM	41 nM	Haloperidol (10 µM)	120 min/RT	Scintillation counting	haloperidol (IC50:75 n M)	40.267	<50% @10 µM
Ca									42.600	<50% @10 µM
Kv	rat cerebral cortex	[125I]a-dendrotoxin	0.01 nM	0.04 nM	a-dendrotoxin (50 nM)	60 min/RT	Scintillation counting	a-dendrotoxin (IC50:0.1 n M)	-11.4	<50% @10 µM
SKCa	rat cerebral cortex	[125I]apamin	0.007 nM	0.007 nM	apamin (100 nM)	60 min/4°C	Scintillation counting	apamin (IC 50:0.0091 n M)	-4.600	<50% @10 µM
Nav	rat cerebral cortex								65.100	
GABA Cl- channel (GABA-gated)	rat cerebral cortex	[35S]TBPS	3 nM	14.6 nM	picrotoxinin (20 µM)	120 min/RT	Scintillation counting	picrotoxinin (IC50:150 n M)	17	<50% @10 µM
Purinergic Channels P2X	rat urinary bladder	"[3H]a,b-MeATP"	3 nM	2.6 nM	"a,b-MeATP (10 µM)"	120 min/4°C	Scintillation counting	"a,b-MeATP (IC 50:3.5 nM)"	14.2333333 3	<50% @10 µM
Purinergic Channels P2Y	rat cerebral cortex	[35S]dATPaS	10 nM	10 nM	dATPaS (10 µM)	60 min/RT	Scintillation counting	dATPaS (IC 50:20 nM)	17.467	<50% @10 µM
Dopamine transporter	human recombinant (CHO cells)	[3H]BTCP	4 nM	4.5 nM	BTCP (10 µM)	120 min/4°C	Scintillation counting	BTCP (IC50 :7.1 nM)	91.200	
Norepinephrine transporter	human recombinant (CHO cells)	[3H]nisoxetine	1 nM	2.9 nM	desipramine (1 µM)	120 min/4°C	Scintillation counting	protriptyline (IC50:5.4 n M)	13.567	<50% @10 µM
Serotonin transporter	human recombinant (CHO cells)	[3H]imipramin e	2 nM	1.7 nM	imipramine (10 µM)	60 min/RT	Scintillation counting	imipramine (IC50:2.1 n M)	-5.2	<50% @10 µM

Table S3. Inhibitory activity of Benzopyran-G1 (SR) on protein targets

	Source	Ligand	Ligand concentration	Kd (nM)	Non specific	Incubation	Detection method	Reference:	Specific Binding 10 µM	% Specific Binding at 10 µM
Adenosine A1	human recombinant (CHO cells)	[3H]DPCPX	1 nM	1.7 nM	DPCPX (1 µM)	60 min/RT	Scintillation counting	DPCPX (IC50:1 nM)	23.333	<50% @10 µM
Adenosine A2A	human recombinant (HEK-293 cells)	[3H]CGS 21680	6 nM	27 nM	NECA (10 µM)	120 min/RT	Scintillation counting	NECA (IC50:28 nM)	-0.967	<50% @10 µM
Adenosine A3	human recombinant (HEK-293 cells)	[125I]AB-MECA	0.15 nM	0.22 nM	IB-MECA (1 µM)	120 min/RT	Scintillation counting	IB-MECA (IC50:0.27 nM)	24.800	<50% @10 µM
Adrenergic α1	rat cerebral cortex	[3H]prazosin	0.25 nM	0.09 nM	prazosin (0.5 µM)	60 min/RT	Scintillation counting	prazosin (IC50:0.182 nM	8.200	<50% @10 µM
Adrenergic α2	rat cerebral cortex	[3H]prazosin	0.25 nM	0.09 nM	prazosin (0.5 µM)	60 min/RT	Scintillation counting	prazosin (IC50:0.182 nM	8.700	<50% @10 µM
Adrenergic β1	rat cerebral cortex	[3H]RX 821002	0.5 nM	0.38 nM	(-)epinephrine (100 µM)	60 min/RT	Scintillation counting	yohimbine (IC50:58.7 nM)	-0.067	<50% @10 µM
Adrenergic β2	human recombinant (HEK-293 cells)	[3H](-)CGP 12177	0.3 nM	0.39 nM	alprenolol (50 µM)	60 min/RT	Scintillation counting	atenolol (IC50:278 nM)	4.500	<50% @10 µM
Angiotensin-II AT1	human recombinant (HEK-293 cells)	"[125I][Sar1,Ile8]-AT-II"	0.05 nM	0.05 nM	angiotensin-II (10 µM)	120 min/37 °C	Scintillation counting	saralasin (IC50:0.63 nM)	-1.167	<50% @10 µM
Angiotensin-II AT2	human recombinant (HEK-293 cells)	[125I]CGP 42112A	0.01 nM	0.01 nM	angiotensin-II (1 µM)	4 hr/37° C	Scintillation counting	angiotensin-II (IC50:0.14 nM	-0.033	<50% @10 µM
Bombesin BB	rat cerebral cortex	[125I][Tyr4] bombesin	0.01 nM	0.71 nM	bombesin (1 µM)	60 min/RT	Scintillation counting	bombesin (IC50:0.205 nM)	-16.833	<50% @10 µM
Benzodiazepine BDZ Peripheral	rat heart	[3H]PK 11195	0.2 nM	1.8 nM	PK 11195 (10 µM)	15 min/RT	Scintillation counting	PK 11195 (IC50:0.98 nM)	26.867	<50% @10 µM
Benzodiazepine BDZ Central	rat cerebral cortex	[3H]flunitrazepam	0.4 nM	2.1 nM	diazepam (3 µM)	60 min/4° C	Scintillation counting	diazepam (IC50:8.6 nM)	-12.533	<50% @10 µM
Bradykinin B2	human recombinant (CHO cells)	[3H]desArg10-KD	0.35 nM	0.085 nM	desArg9[Leu8]-BK (10 µM)	60 min/RT	Scintillation counting	desArg10-KD (IC50:0.77 nM)	-3.3	<50% @10 µM
Calcitonin gene-related peptide CGRP	human recombinant (CHO cells)	[125I]hCGRPa	0.03 nM	0.06 nM	hCGRPa (1 µM)	90 min/RT	Scintillation counting	hCGRPa (IC50:0.058 nM)	-13.000	<50% @10 µM
Canabinoid CB1	human recombinant (CHO cells)	[3H]CP 55940	0.5 nM	3.5 nM	WIN 55212-2 (10 µM)	120 min/37 °C	Scintillation counting	CP 55940 (IC50:0.58 nM)	-5.300	<50% @10 µM

"Cholecystokinin CCK1 (CCKA), "	human recombinant (CHO cells)	[125I]CCK-8s	0.08 nM	0.24 nM	CCK-8s (1 µM)	60 min/RT	Scintillation counting	CCK-8s (IC50:0.1 nM)	-2.133	<50% @10 µM
Cholecystokinin CCK2 (CCKB)	human recombinant (CHO cells)	[125I]CCK-8s	0.08 nM	0.054 nM	CCK-8s (1 µM)	60 min/RT	Scintillation counting	CCK-8s (IC50:0.098 nM	0.733	<50% @10 µM
Dopamine D1	human recombinant (CHO cells)	[3H]SCH 23390	0.3 nM	0.2 nM	SCH 23390 (1 µM)	60 min/RT	Scintillation counting	SCH 23390 (IC50:0.242 nM)	11.400	<50% @10 µM
Dopamine D2S	human recombinant (HEK-293 cells)	[3H]methyl-spiperone	0.3 nM	0.15 nM	(+)butaclamol (10 µM)	60 min/RT	Scintillation counting	(+)butaclamol (IC50:1.64 nM)	3.533	<50% @10 µM
Dopamine D3	human recombinant (CHO cells)	[3H]methyl-spiperone	0.3 nM	0.085 nM	(+)butaclamol (10 µM)	60 min/RT	Scintillation counting	(+)butaclamol (IC50:1.4 nM)	-2.533	<50% @10 µM
Dopamine D4.4	human recombinant (CHO cells)	[3H]methyl-spiperone	0.3 nM	0.19 nM	(+)butaclamol (10 µM)	60 min/RT	Scintillation counting	clozapine (IC50:46.5 nM	-2.500	<50% @10 µM
Dopamine D5	human recombinant (GH4 cells)	[3H]SCH 23390	0.3 nM	0.25 nM	SCH 23390 (10 µM)	60 min/RT	Scintillation counting	SCH 23390 (IC50:0.39 nM)	-2.900	<50% @10 µM
Endothelin ETA	human recombinant (CHO cells)	[125I]endothelin-1	0.03 nM	0.03 nM	endothelin-1 (100 nM)	120 min/37 °C	Scintillation counting	endothelin-1 (IC50:0.038 nM	10.900	<50% @10 µM
Endothelin ETB	human recombinant (CHO cells)	[125I]endothelin-1	0.03 nM	0.04 nM	endothelin-1 (0.1 µM)	120 min/37 °C	Scintillation counting	endothelin-3 (IC50:0.03 nM)	-13.500	<50% @10 µM
GABA (non-selective)	rat cerebral cortex	[3H]GABA	10 nM	15 nM	GABA (100 µM)	60 min/RT	Scintillation counting	GABA (IC50:38 nM)	-0.200	<50% @10 µM
GAL1	human recombinant (HEK-293 cells)	[125I]galanin	0.1 nM	0.1 nM	galanin (1 µM)	60 min/RT	Scintillation counting	galanin (IC50:0.35 nM	-3.400	<50% @10 µM
GAL2	human recombinant (CHO cells)	[125I]galanin	0.05 nM	0.63 nM	galanin (1 µM)	120 min/RT	Scintillation counting	galanin (IC50:0.3 nM	-8.167	<50% @10 µM
PDGF	Balb/c 3T3 cells	[125I]PDGF BB	0.03 nM	0.15 nM	PDGF BB (10 nM)	180 min/4°C	Scintillation counting	PDGF BB (IC50:0.093 nM)	-23.400	<50% @10 µM
Chemokines CCR1	human recombinant (HEK-293 cells)	[125I]MIP-1a	0.01 nM	0.02 nM	MIP-1a (100 nM)	120 min/RT	Scintillation counting	MIP-1a (IC50:0.04 nM)	-10.967	<50% @10 µM
Chemokines CXCR2 (IL-8B)	human recombinant (HEK-293 cells)	[125I]IL-8	0.025 nM	0.022 nM	IL-8 (30 nM)	60 min/RT	Scintillation counting	IL-8 (IC50:0.115 nM)	1.767	<50% @10 µM
Histamine H1	human recombinant (HEK-293 cells)	[3H]pyrilamine	1 nM	1.7 nM	pyrilamine (1 µM)	60 min/RT	Scintillation counting	pyrilamine (IC50:2.2 nM	-1.000	<50% @10 µµM

Histamine H2	human recombinant (CHO cells)	[125I]APT	0.075 nM	2.9 nM	tiotidine (100 µM)	120 min/RT	Scintillation counting	cimetidine (IC50:350 nM)	83.600	
Melanocortin MC4	human recombinant (CHO cells)	[125I]NDP-a-MSH	0.05 nM	0.54 nM	NDP-a-MSH (1 µM)	120 min/37 °C	Scintillation counting	NDP-a-MSH (IC50: 0.23 nM)	5.533	<50% @10 µM
Melatonin MT1	human recombinant (CHO cells)	[125I]2-iodomelatonin	0.01 nM	0.04 nM	melatonin (1 µM)	60 min/RT	Scintillation counting	melatonin (IC50:0.3 nM)	22.900	<50% @10 µM
Muscarinic M1	human recombinant (CHO cells)	[3H]pirenzepine	2 nM	13 nM	atropine (1 µM)	60 min/RT	Scintillation counting	pirenzepine (IC50:22 nM)	4.200	<50% @10 µM
Muscarinic M2	human recombinant (CHO cells)	[3H]AF-DX 384	2 nM	4.6 nM	atropine (1 µM)	60 min/RT	Scintillation counting	methoctramine (IC50:32 nM)	-1.833	<50% @10 µM
Muscarinic M3	human recombinant (CHO cells)	[3H]AF-DX 384	2 nM	4.6 nM	atropine (1 µM)	60 min/RT	Scintillation counting	methoctramine (IC50:32 nM)	20.167	<50% @10 µM
Muscarinic M4	human recombinant (CHO cells)	[3H]4-DAMP	0.2 nM	0.32 nM	atropine (1 µM)	60 min/RT	Scintillation counting	4-DAMP (IC50:0.53 nM)	13.933	<50% @10 µM
Muscarinic M5	human recombinant (CHO cells)	[3H]4-DAMP	0.3 nM	0.3 nM	atropine (1 µM)	60 min/RT	Scintillation counting	4-DAMP (IC50:0.52 nM	8.400	<50% @10 µM
Neurokinin NK1	U373MG uppsala	[125I]-Substance P LYS3	0.05 nM	0.04 nM	"[Sar9,Met(O2)11]-SP (1 µM)"	30 min/RT	Scintillation counting	"[Sar9,Met(O2)11]-SP (IC50:0.29 nM)"	64.167	
Neurokinin NK2	human recombinant (CHO cells)	[125I]NKA	0.1 nM	0.12 nM	[Nleu10]-NKA (4-10) (300 nM)	60 min/RT	Scintillation counting	[Nleu10]-NKA (4-10) (IC50:2.7 nM)	22.167	<50% @10 µM
Neurokinin NK3	human recombinant (CHO cells)	[3H]SR 142801	0.4 nM	0.47 nM	SB 222200 (10 µM)	120 min/RT	Scintillation counting	SB 222200 (IC50:8.8 nM)	2.267	<50% @10 µM
Neuropeptide-Y Y1	SK-N-MC cells (endogenous)	[125I]peptide YY	0.025 nM	0.06 nM	NPY (1 µM)	120 min/37 °C	Scintillation counting	NPY (IC50:0.079 nM)	-11.733	<50% @10 µM
Neuropeptide-Y Y2	KAN-TS cells	[125I]peptide YY	0.015 nM	0.01 nM	NPY (1 µM)	60 min/37 °C	Scintillation counting	NPY (IC50:0.0555 nM)	-10.500	<50% @10 µM
Neurotensin NTS1 (NT1)	human recombinant (CHO cells)	[125I]Tyr3-neurotensin	0.05 nM	0.22 nM	neurotensin (1 µM)	60 min/4°C	Scintillation counting	neurotensin (IC50:0.3 nM)	-15.100	<50% @10 µM
Opioid δ2	human recombinant (CHO cells)	[3H]DADLE	0.5 nM	0.73 nM	naltrexone (10 µM)	120 min/RT	Scintillation counting	DPDPE (IC50:1.8 nM)	1.900	<50% @10 µM
Opioid Kappa	rat recombinant (CHO cells)	[3H]U 69593	1 nM	2 nM	naloxone (10 µM)	60 min/RT	Scintillation counting	U 50488 (IC50:0.84 nM)	31.267	<50% @10 µM
Opioid µ	human recombinant (HEK-293 cells)	[3H]DAMGO	0.5 nM	0.35 nM	naloxone (10 µM)	120 min/RT	Scintillation counting	DAMGO (IC50:0.537 nM	12.767	<50% @10 µM

NOP (ORL1)	human recombinant (HEK-293 cells)	[3H]nociceptin	0.2 nM	0.4 nM	nociceptin (1 µM)	60 min/RT	Scintillation counting	nociceptin (IC50:0.5 nM)	13.200	<50% @10 µM
PCP	rat cerebral cortex	[3H]TCP	10 nM	13 nM	MK 801 (10 µM)	120 min/37 °C	Scintillation counting	MK 801 (IC50:5.3 nM)	-11.500	<50% @10 µM
EP2	human recombinant (HEK-293 cells)	[3H]PGE2	3 nM	3 nM	PGE2 (10 µM)	120 min/RT	Scintillation counting	PGE2 (IC50: 1.7 nM)	22.400	<50% @10 µM
EP4	human recombinant (HEK-293 cells)	[3H]PGE2	0.5 nM	0.3 nM	PGE2 (10 µM)	120 min/RT	Scintillation counting	PGE2 (IC50: 0.38 nM)	7.367	<50% @10 µM
Prosanoid IP (PGI2)	human recombinant (HEK-293 cells)	[3H]iloprost	6 nM	8 nM	iloprost (10 µM)	60 min/RT	Scintillation counting	iloprost (IC50:22 nM)	21.733	<50% @10 µM
5-HT1A	human recombinant (HEK-293 cells)	[3H]8-OH-DPAT	0.3 nM	0.5 nM	8-OH-DPAT (10 µM)	60 min/RT	Scintillation counting	8-OH-DPAT (IC50: 0.36 nM)	24.167	<50% @10 µM
5-HT1B	rat cerebral cortex	[125I]CYP (+ 30 µM isoproterenol)	0.1 nM	0.16 nM	serotonin (10 µM)	120 min/37 °C	Scintillation counting	serotonin (IC50:10 nM)	-14.133	<50% @10 µM
5-HT2A	human recombinant (HEK-293 cells)	[3H]ketanserin	0.5 nM	0.6 nM	ketanserin (1 µM)	60 min/RT	Scintillation counting	ketanserin (IC50:0.73 nM	46.400	<50% @10 µM
5-HT2B	human recombinant (CHO cells)	[125I](±)DOI	0.2 nM	0.2 nM	(±)DOI (1 µM)	60 min/RT	Scintillation counting	(±)DOI (IC50:5 nM	-20.133	<50% @10 µM
5-HT2C	human recombinant (HEK-293 cells)	[3H]mesulergine	1 nM	0.5 nM	RS 102221(10 µM)	120 min/37 °C	Scintillation counting	RS 102221 (IC50:3.1 nM)	4.400	<50% @10 µM
5-HT3	human recombinant (CHO cells)	[3H]BRL 43694	0.5 nM	1.15 nM	MDL 72222 (10 µM)	120 min/RT	Scintillation counting	MDL 72222 (10 nm)	-7.233	<50% @10 µM
5-HT5a	human recombinant (HEK-293 cells)	[3H]LSD	1.5 nM	1.5 nM	serotonin (100 µM)	120 min/37 °C	Scintillation counting	serotonin (IC50:120 nM)	16.267	<50% @10 µM
5-HT6	human recombinant (CHO cells)	[3H]LSD	2 nM	1.8 nM	serotonin (100 µM)	120 min/37 °C	Scintillation counting	serotonin (IC50:150 nM)	28.833	<50% @10 µM
5-HT7	human recombinant (CHO cells)	[3H]LSD	4 nM	2.3 nM	serotonin (10 µM)	120 min/RT	Scintillation counting	serotonin (IC50:0.45 nM	19.800	<50% @10 µM
Somatostatin sst	AtT-20 cells	[125I]Tyr11-somatostatin-14	0.05 nM	0.08 nM	somatostatin-14 (300 nM)	60 min/37 °C	Scintillation counting	somatostatin-14 (IC50:0.2 nM)	-3.4	<50% @10 µM
Vasoactive intestinal peptide	human recombin	[125I]PACAP1-27	0.015 nM	0.092 nM	PACAP1-27 (100 nM)	120 min/RT	Scintillation counting	PACAP1-38 (IC50:0.08 nM)	-28.16666667	<50% @10 µM

PAC1 (PACAP)	ant (CHO cells)									
Vasoactive intestinal peptide VPAC1 (VIP1)	human recombinant (CHO cells)	[125I]VIP	0.04 nM	0.05 nM	VIP (1 µM)	60 min/RT	Scintillation counting	VIP (IC50:0.28 nM)	-8.4	<50% @10 µM
Vasopressin (V1a)	human recombinant (CHO cells)	[3H]AVP	0.3 nM	0.5 nM	AVP (1 µM)	60 min/RT	Scintillation counting	"[d(CH2)51,Tyr(Me)2]-AVP (IC50:0.89 nM)"	5.766666667	<50% @10 µM
Steroid nuclear receptors GR	IM-9 cells (cytosol)	[3H]dexamethasone	1.5 nM	1.5 nM	triamcinolone (10 µM)	6 hr/4°C	Scintillation counting	dexamethasone (IC50:4.3 nM)	3.067	<50% @10 µM
Non-steroid nuclear receptors PPAR?	human recombinant (E. coli)	[3H]rosiglitazone	5 nM	5.7 nM	rosiglitazone (10 µM)	120 min/4°C	Scintillation counting	rosiglitazone (IC50:11 nM)	58.300	
TNF-α	U-937 cells	[125I]TNF-a	0.1 nM	0.05 nM	TNF-a (10 nM)	120 min/4°C	Scintillation counting	TNF-a (IC50:0.1 nM)	2.033	<50% @10 µM
sigma (non-selective)	Jurkat cells (endogenous)	[3H]DTG	10 nM	41 nM	Haloperidol (10 µM)	120 min/RT	Scintillation counting	haloperidol (IC50:75 nM)	20.167	<50% @10 µM
Ca									28.633	<50% @10 µM
Kv	rat cerebral cortex	[125I]a-dendrotoxin	0.01 nM	0.04 nM	a-dendrotoxin (50 nM)	60 min/RT	Scintillation counting	a-dendrotoxin (IC50:0.1 nM)	-18.73333333	<50% @10 µM
SKCa	rat cerebral cortex	[125I]apamin	0.007 nM	0.007 nM	apamin (100 nM)	60 min/4°C	Scintillation counting	apamin (IC50:0.0091 nM)	-16.667	<50% @10 µM
Nav	rat cerebral cortex								68.800	
GABA Cl- channel (GABA-gated)	rat cerebral cortex	[35S]TBPS	3 nM	14.6 nM	picrotoxinin (20 µM)	120 min/RT	Scintillation counting	picrotoxinin (IC50:150 nM)	32.83333333	<50% @10 µM
Purinergic Channels P2X	rat urinary bladder	"[3H]a,b-MeATP"	3 nM	2.6 nM	"a,b-MeATP (10 µM)"	120 min/4°C	Scintillation counting	"a,b-MeATP (IC50:3.5 nM)"	2.233333333	<50% @10 µM
Purinergic Channels P2Y	rat cerebral cortex	[35S]dATPaS	10 nM	10 nM	dATPaS (10 µM)	60 min/RT	Scintillation counting	dATPaS (IC50:20 nM)	7.900	<50% @10 µM
Dopamine transporter	human recombinant (CHO cells)	[3H]BTCP	4 nM	4.5 nM	BTCP (10 µM)	120 min/4°C	Scintillation counting	BTCP (IC50:7.1 nM)	44.600	<50% @10 µM
Norepinephrine transporter	human recombinant (CHO cells)	[3H]nisoxetine	1 nM	2.9 nM	desipramine (1 µM)	120 min/4°C	Scintillation counting	protriptyline (IC50:5.4 nM)	5.800	<50% @10 µM
Serotonin transporter	human recombinant (CHO cells)	[3H]imipramine	2 nM	1.7 nM	imipramine (10 µM)	60 min/RT	Scintillation counting	imipramine (IC50:2.1 nM)	-4.8	<50% @10 µM

Table S4. The potency of inhibitory activity of Benzopyran-G1 on protein targets

Assay	IC_{50} (M)	K_i	nH
α1 (non-selective) (antagonist radioligand	1.3E-05	3.5E-06	1.3
Dopamine transporter (h) (antagonist radioligand)	1.0E-06	5.5E-07	1

Table S5. Rat PK, IV/PO cross over and brain penetration data

	Benzopyran-G1	
**In vitro ADME**	**Solubility (µM)**	
	**Rat Plasma Protein Binding (PPB)**	89%
	**Human PPB**	97%
	**Dog PPB**	94%
**Rat Pharmacokinetics, 1mg/kg iv.**	**Rat iv. AUC∞ (ng.hr/ml)**	4953
	**Co (ng/ml)**	3941
	**Vd (L/kg)**	1%
	**CL (ml/min/kg)**	3.39
**Rat Pharmacokinetics, 5mg/kg po.**	**Rat p.o. AUC∞ (ng.hr/ml)**	16802 *
	**Rat p.o. AUCt (ng.hr/ml)**	6416
	**C_{MAX} (ng/ml)**	1191
	**CL (ml/min/kg)**	3.3
	**Brain at Tmax (ng/ml)**	121
	**CNS Penetration (B:P)**	20%
	**T1/2 (hrs)**	12.2
	**Oral bioavailability F%**	32%

APPENDIX 1 - MAINTENANCE OF CELL LINES STABLY EXPRESSING ION CHANNELS

Experiments were performed on a number of cell lines stably expressing a variety of ion channels. A cell line expressing Kir3.4 was created in-house using standard techniques of sub-cloning an appropriate DNA sequences into a mammalian expression vector together with a gene for specific antibiotic resistance (see **Table S6**). Expression constructs were transfected into HEK293 cells prior to selection and sub-cloning before finally being assayed for current expression and validated electrophysiologically. All other cell lines were sourced externally (Kir3.1/3.4, Kir6.2/SUR2A [Prof. Andrew Tinker, UCL], Nav1.5 [UPenn]; hERG [Cytomyxs or Cytocentrics]; Kv1.5 [BSYS] and Kv4.3, Kir2.1, Kv7.1/KCNE1 [bioFocusDPI]).

All cell lines were grown to adhere to T-175 flasks and passaged using an enzymatic agent (Accutase, ICT, Inc.). All cell lines were plated out onto small sterilized glass coverslips in 35 mm x 10 mm Polystyrene Petri dishes (Corning) containing an appropriate medium, supplemented with 10 % foetal bovine serum (Hyclone Perbio). The cells were incubated at 37 or 30 °C (5 % CO_2) for 1-4 days prior to any electrophysiological study.

Table S6. Cell Culture Conditions

	Media	Serum	Selection Antibiotic	Other Supplements
**Kv1.5**	JRH Ex-Cell 302 (JRH Biosciences)	-	2 µg / ml Blasticidin (Invitrogen)	8 mM L-Glutamine (Gibco), 1% HT Supplement
**Kv1.3**	Nutrient mixture IMDM#21980	10 % Fatalclone II (Hyclone Perbio)	400 µg / ml Geneticin (Gibco)	1% HT Supplement
**Kv4.3**	Nutrient mixture F12 (Gibco)	10 % Fatalclone II (Hyclone Perbio)	500 µg / ml Geneticin (Gibco)	1% NEAA
**Nav1.5**	Nutrient mixture F12 (Gibco)	10 % Fatalclone II (Hyclone Perbio)	500 µg / ml Geneticin (Gibco)	-
**Kir2.1**	MEM #31095	10 % Fatalclone II (Hyclone Perbio)	300 µg / ml Hygromycine B	1% NEAA
**Kir3.x**	MEM #31095	10% Invitrogene FBS #16000	182 µg / ml Zeocin	-
**hERG**	Nutrient mixture DMEM	10 % Fatalclone II (Hyclone Perbio)	200 µg / ml Geneticin (Gibco)	1% L-Glutamine (Gibco), 1% Na Pyruvate
**Cav1.2**	Nutrient mixture DMFM F12 #31331	10 % Fatalclone II (Hyclone Perbio)	500 µg / ml Geneticin (Gibco)	-

APPENDIX 2 - EXTERNAL ASSAY SOLUTION COMPOSITION

Table S7-1. Table detailing composition (in mM) of external experimental assay solutions

	Kv1.5<br>Kv4.3<br>Kv1.7<br>(mM)	Kir6.2<br>(mM)	Kv7.1<br>(mM)	Nav1.5<br>(mM)	hERG<br>(mM)	Kir2.1<br>Kir3.x<br>(mM)	0 mM K^+<br>(mM)
**NaCl**	150	150	-	140	140	150	150
**KCl**	10	10	-	5	4	10	0
**NMDG**		-	140	-	-	-	-
**K-gluconate**	-	-	5	-	-	-	-
**$CaCl_2$**	3	3	2	1	2	3	3
**$MgCl_2$**	1	1	1	1.2	1	1	1
**Glucose**	-	-	5	11.1	10	-	-
**Sucrose**	-	-	50	-	-	-	-
**HEPES**	10	10	10	5	5	10	10
**pH**	7.4	7.4	7.4	7.4	7.4	7.4	7.4
**pH adjusted**	NaOH	NaOH	CH_3SO_3H	NaOH	NaOH	NaOH	NaOH

Table S7-2. Table detailing composition (in mM) of external experimental assay solutions

	**Cav1.2 resting solution, RPS (mM)**	**Cav1.2 Dye-loading solution (mM)**	**Cav1.2 stimulus solution (mM)**
**NaCl**	140	140	90
**KCl**	5	5	55
**$CaCl_2$**	5	5	5
**$MgCl_2$**	1	1	1
**Glucose**	10	10	10
**Fluo4 NW**	-	10 mg in 12 ml RPS	-
**Probenecid**	2.5	2.5	2.5
**FPL-64176**	-		6.1^{-4}-
**HEPES**	15	15	15
**pH**	7.4	7.4	7.4
**pH adjusted**	NaOH	NaOH	NaOH

APPENDIX 3 – INTERNAL PIPETTE SOLUTION COMPOSITIONS

Table S8. Table detailing composition (in mM) of internal experimental pipette solutions

	Kv1.5/Kv4.3/Kv1.7 (mM)	Kv7.1 (mM)	Kir2.1 (mM)	Kir3.x (mM)	Kir6.2 (mM)	Nav1.5 (mM)	hERG (mM)
**KCl**	20	-	110	110	125	-	130
**K-gluconate**	-	90	-	-	-	-	-
**KF**	90	20	-	-	-	-	-
**CsF**	-	-	-	-	-	120	-
**NaCl**	10	-	20	20	10	15	-
**Na-gluconate**	-	10	-	-	-	-	-
**$MgCl_2$**	1	1	1	1	1.1	-	1
**$CaCl_2$**	-	-	-	-	0.5	-	-
**Mg-ATP**	-	-	5	5	3.15	-	5
**Na_2-ATP**	5	5	-	-	-	-	-
**HEPES**	10	10	10	10	7.5	10	10
**Sucrose**	10	20	-	-	-	-	-
**EGTA**	10	10	5	5	7.5	10	5
**EDTA**	-	10	-	-	-	-	-
**GTP-γS**	-	-	-	0.9 (Added fresh)	-	-	-
**pH**	7.2	7.2	7.2	7.2	7.2	7.25	7.2
**pH adjusted**	KOH	KOH	KOH	KOH	KOH	CsOH	KOH

APPENDIX 4- EQUATIONS

$$\%Inhibition = (1 - I_{Intervention}/I_{Control}) \times 100$$

Equation 1 – Percentage Inhibition
Inhibition of current (or leak-subtracted current) was calculated from steady-state current recorded in the absence ($I_{Control}$) and presence of test-article ($I_{Intervention}$).

$$\% Inhibition = Y_{Min} + \left\{ \frac{(Y_{Max} - Y_{Min})}{1 + 10^{(LogIC_{50} - x)n_H}} \right\}$$

Equation 2 – Concentration-response curve
Concentration-response data were fitted with a sigmoidal function of the form above, where Y_{Min} and Y_{Max} represent maximum and minimum current inhibition which were constrained to 100 and 0 respectively and x is the Log[DRUG (M)] based on GraphPad Prism analysis. % inhibition was calculated as in Equation 1.
